# Supplementary material for: Experimental and machine learning-based exploration of repurposed drugs reveals chemical features underlying phospholipidosis
Source: Patterns (N Y). 2026 Feb 6;7(4):101453. doi: 10.1016/j.patter.2025.101453 (PMC13083723; doi:10.1016/j.patter.2025.101453)
Supplement: Document S1. Figures S1–S5 and Table S1 [file mmc1.pdf]

Patterns, Volume *7*

Supplemental information

Experimental and machine learning-based exploration of repurposed drugs reveals chemical features underlying phospholipidosis

Maria Kuzikov, Adelinn Kalman, Reagon Karki, Jeanette Reinshagen, Johanna Huchting, Kun Qian, Hanna Axelsson, Marianna Tampere, Päivi Östling, Brinton Seashore-Ludlow, Yojana Gadiya, Philip Gribbon, and Andrea Zaliani

Supplementary material

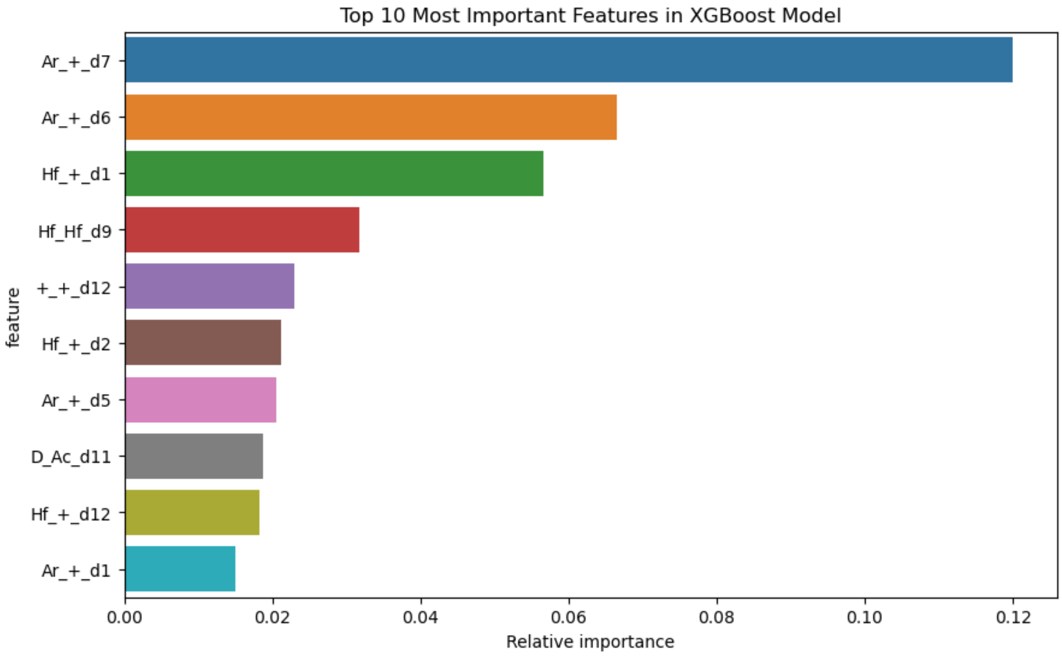

Figure S1: Bar chart reporting the top 10 most influential descriptors for the XGBoost model. The top features shown in this figure are the ErG annotations. In this notation, for instance, a descriptor like "Ar_+_d7" means that bits are set to 1 when an aromatic centroid lies seven bonds distant from a positive charge. Hf identifies a hydrophobic group (a collection of at least three carbon atoms), while "D" and "Ac" identify hydrogen-bond donors and acceptors, respectively.

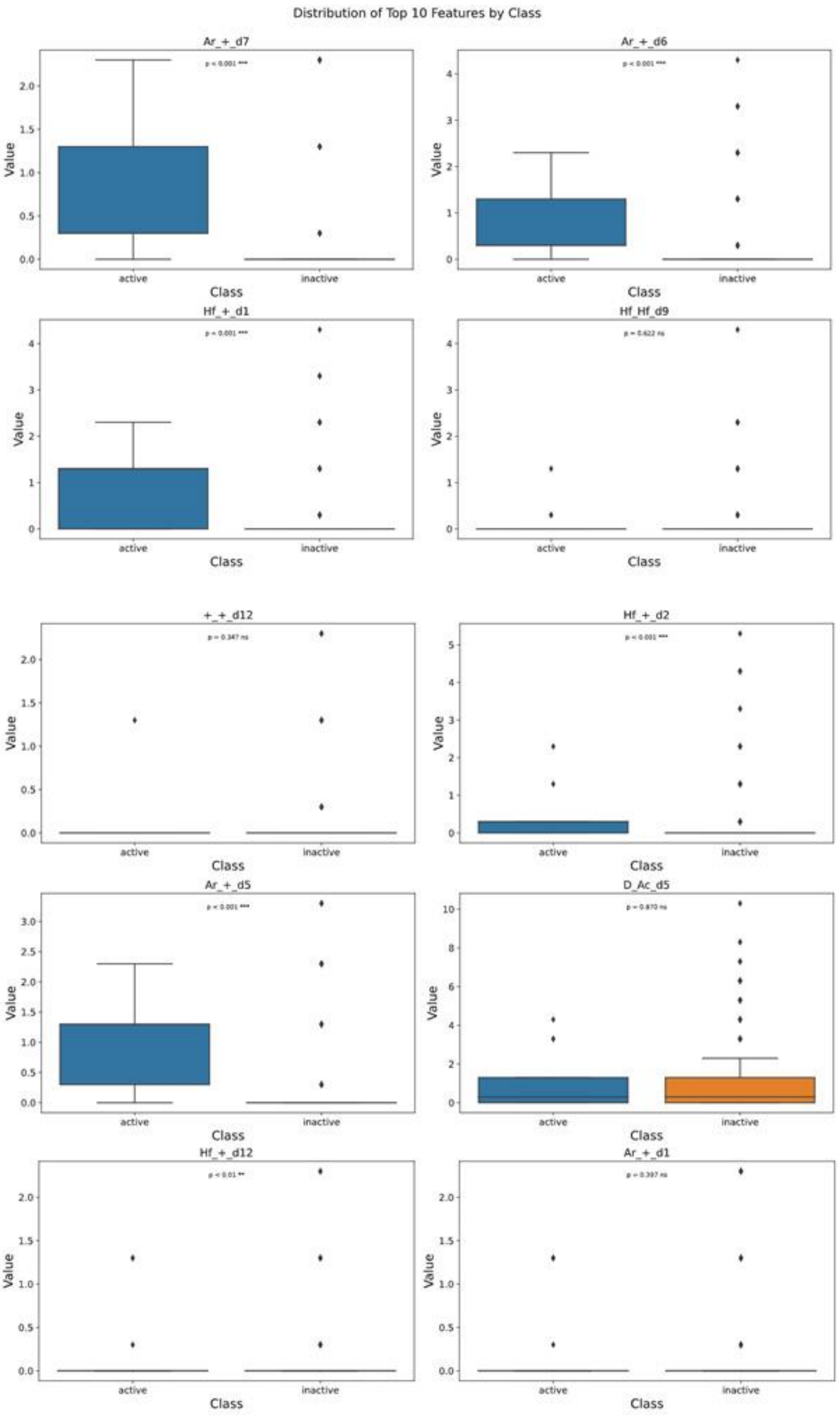

Figure S2: Distribution of the top10 most influential ErG features across the two classes with enhanced box plots. To understand the distribution across the two classes namely Active and Inactive in the original dataset. In almost all features chosen by the XGBoost model, Actives show higher values than Inactives. Significance p-values are given.

Table S1. PLD EC_{50} determination of anti-SARS-CoV-2 CPE active compounds, correlation to available CPE EC_{50}. PLD effect is normalized to amiodarone PLD induction effect at 10 µM, set to 100 %, DMSO is used as a negative control, set to 0 % PLD induction effect. Anti-SARS-CoV-2 CPE Data points are measured at 20 µM for infection in CaCo2 cells and at 10 µM for infection in Vero—E6 cells. Data points represent triplicate measurements. Empty fields: no data available

Effect in %								
**Name**	**20 µM**	**10 µM**	**5 µM**	**EC50 µM**	**Vero-E6 CPE inhibition %**	**Vero-E6 EC_{50} µM**	**CaCo2 CPE inhibition %**	**CaCo2 EC_{50} µM**
DDR1-IN-1	21.77	75.83	55.73	>20	-0.37		77.58	
TAK-960	-40.84	151.30	132.37	1.47	2.67		91.64	
SB-216641	-42.60	104.74	93.22	1.19	-0.22		57.65	
Lylamine	-41.01	133.55	104.48	>20	-0.36		76.43	
SB-743921	-43.61	223.62	171.82	2.49	-0.36		78.89	
DR-4485	-16.80	201.85	94.34	>20	-0.26		82.83	
Dexniguldipine	127.40	114.68	83.07	2.48	-0.15		97.57	
Cobimetinib	133.45	134.56	112.38	1.34	-0.31		61.58	
Lomitapide	-28.65	169.31	82.84	>20	-0.39		77.37	
Afatinib	146.21	248.65	128.55	>20	-0.39		60.16	
KHK-IN-1	toxic	toxic	toxic	toxic	toxic	toxic	toxic	toxic
NNC-55-0396	-43.08	154.81	87.06	6.28	0	8.21	58.09	
GR-127935	-40.19	161.77	136.46	1.12	-0.76		53.73	
Ceritinib	137.34	116.10	91.93	2.18	-0.35		59.98	
TAS-103	121.21	172.31	135.39	2.29	-0.08		92.56	
lidoflazine	129.06	80.16	65.28	5.51	0.5		95.92	17.39
tetrindole	-33.00	133.70	133.51	9.59	-0.34		75.19	
PD-161570	36.18	118.17	163.59	0.63	12.35	144.52	108.10	
U-18666A	112.55	98.22	84.91	1.77	9.72	1.15	77.44	
JHW-007	86.69	112.39	115.34	0.71	-0.32		93.14	
casin	134.63	193.97	116.35	3.31	-0.24	14.15	71.19	
chlorprothixene hydrochloride	191.97	112.70	124.38	2.36	0.27		55.65	
mefloquine hydrochloride	174.50	187.70	102.25	1.48	-0.32		93.28	14.15
NNC-05-2090	118.98	178.14	128.41	4.91	-0.32		71.83	
halofantrine HCl	113.55	86.98	79.75	1.66	0.17		91.38	
quinacrine hydrochloride	-13.21	141.12	145.20	1.55	-0.23		90.28	
amodiaquine dihydrochloride	186.68	101.86	88.75	>20	20.16	21.51	78.80	
norfluoxetine	197.87	111.51	92.13	1.96	-0.11		94.99	
metergoline	102.77	73.02	61.00	>20	0.05		54.65	
SNAP-5089	24.87	78.37	66.55	2.01	-0.12		72.63	
BiBX-1382	-0.64	75.46	85.75	0.61	0.28		79.29	
BIBU-1361	-42.53	180.15	160.51	0.63	-0.24		78.51	
tecalcet	101.68	74.29	59.63	2.6	-0.13		78.10	
tafenoquine	toxic	toxic	toxic	toxic	toxic	toxic	toxic	toxic
loperamide hydrochloride	86.14	75.44	55.03	2.74	5.03		58.93	

3'-fluorobenzylspiperone	8.92	41.30	61.76	10.3	72.81	7.66	102.38	
SR-33805 oxalate	137.08	86.07	54.44	9.26	0.02		67.72	
entrectinib (RXDX-101)	toxic	toxic	toxic	toxic	toxic	toxic	toxic	toxic
mibefradil	2.41	80.50	62.04	2.45	-0.26		52.45	
RG7112 (RO50455337)	88.45	141.41	109.12	2.56	6.32		88.52	
Vandetanib (ZD6474)	-32.66	136.39	128.97	1.05	0.29		51.24	
CID-2011756	55.98	31.32	27.66	>20	0.15		96.96	
ispinesib (SB-715992)	-43.20	205.19	206.65	1.81	-0.29		91.86	
AMG-073 HCl (cinacalcet hydrochloride)	252.97	79.48	70.80	18.58	-0.15		51.60	
masitinib (AB1010)	53.29	45.24	43.15	2.57	37	11.9	107.90	
Pelitinib (EKB-569)	100.63	85.52	76.13	>20	-0.31		83.39	
BMS-833923 (XL139)	-39.59	59.08	72.20	1.11	-0.37		68.77	
DBeQ	-36.11	88.88	73.45	1.19	-0.3		100.89	

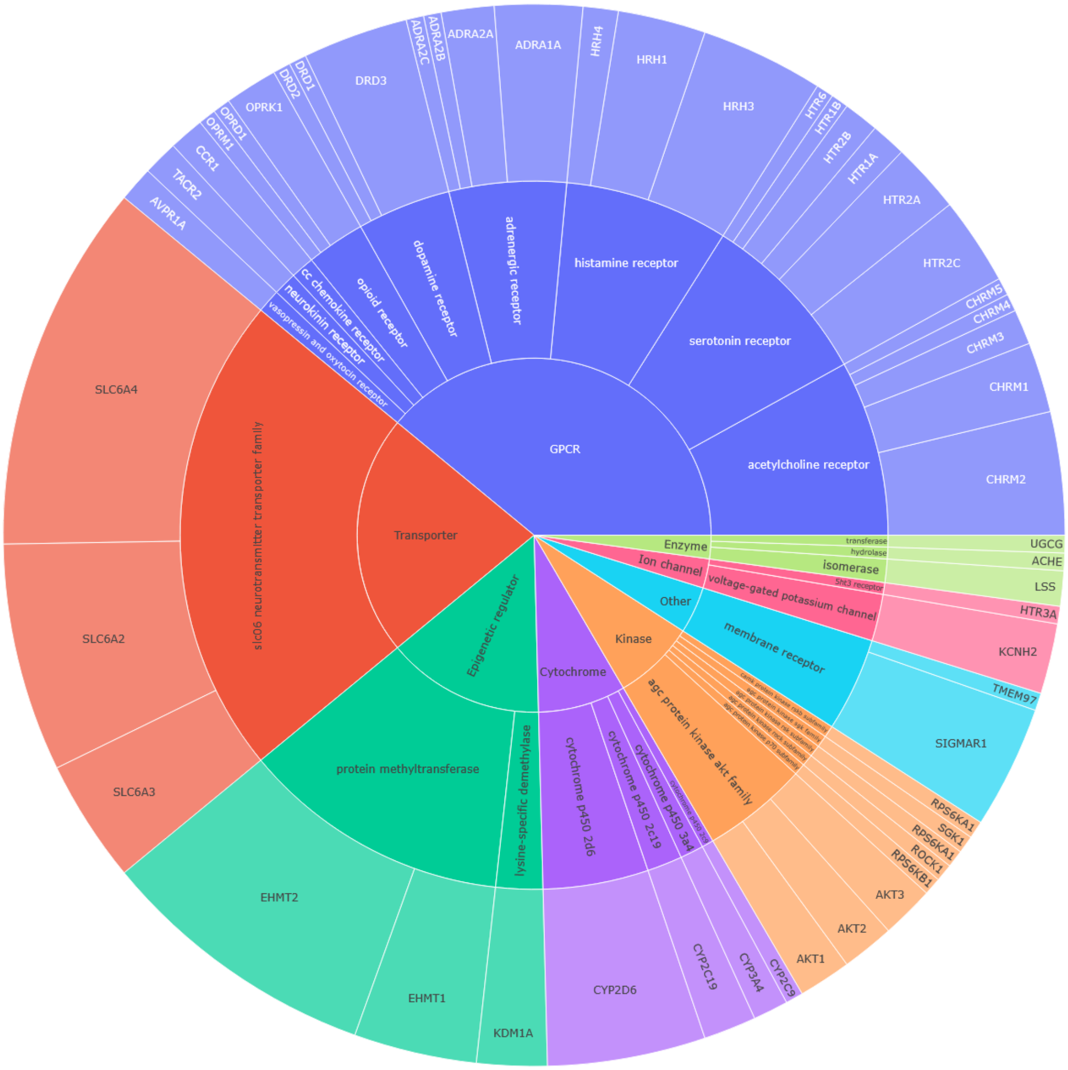

Figure S3. Distribution of cellular target for hit compounds identified in the primary screen for PLD induction in Vero-E6 and A549-ACE2. Annotation according to ChEBML v34. The 58 active compounds were searched into the ChEMBL database for identification of previously reported binding or functional assay experiments with other known targets. To achieve this, we used the pythonic functions provided by the KGG workflow (https://github.com/Fraunhofer-ITMP/kgg). The resulting output contains informtion about each compound's measured value (eg. Log Ki, IC50, AC50, etc.) for a specific target in a particular assay experiment (Supplementary file "Annotated_targets_to_PLD_hits").

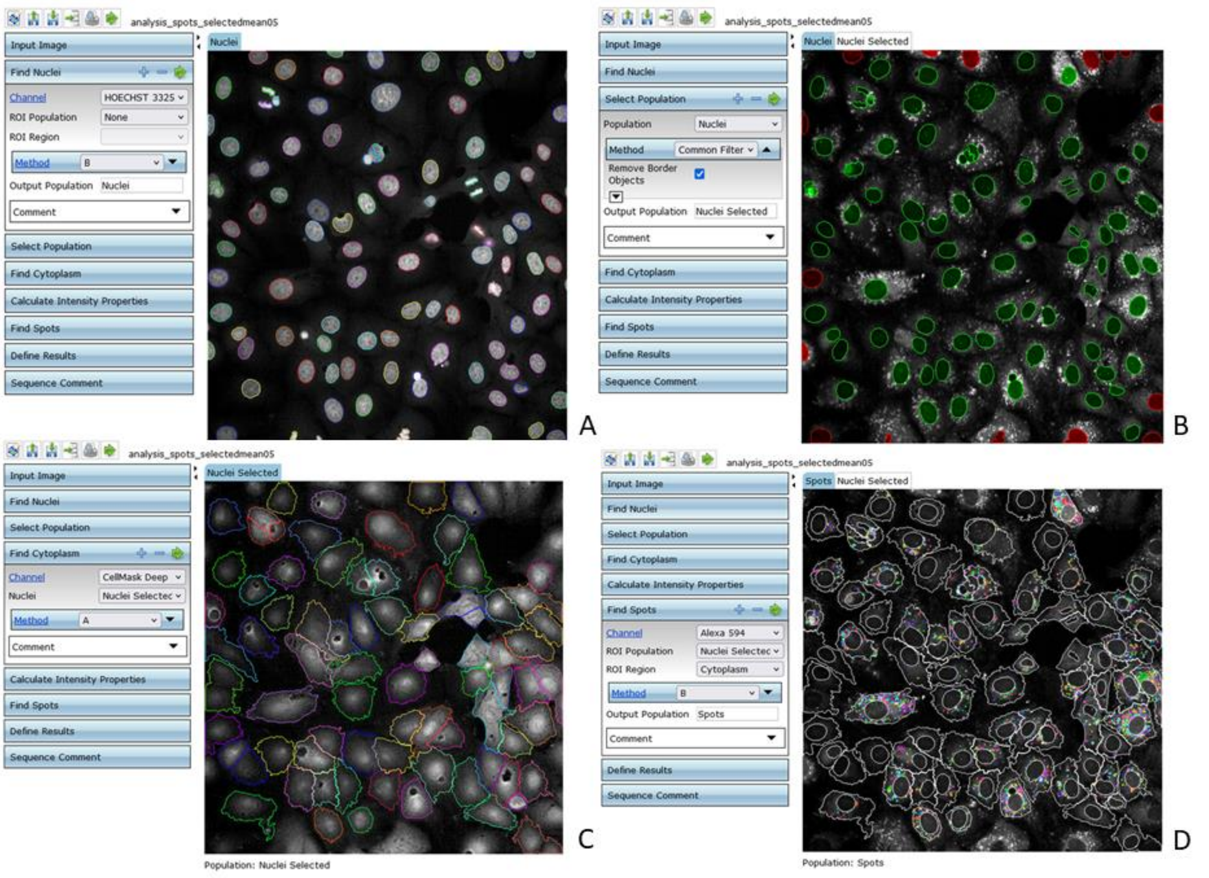

Figure S4: Workflow of DIPL data analysis in Columbus Image Analysis Software. Nuclei identification (A) Removement of border objects (nuclei are completely displayed) = “selected population” (B) Cytoplasm identification in the selected population (C) Calculation of DIPL signal in the cytoplasm: Identification of spots (D).

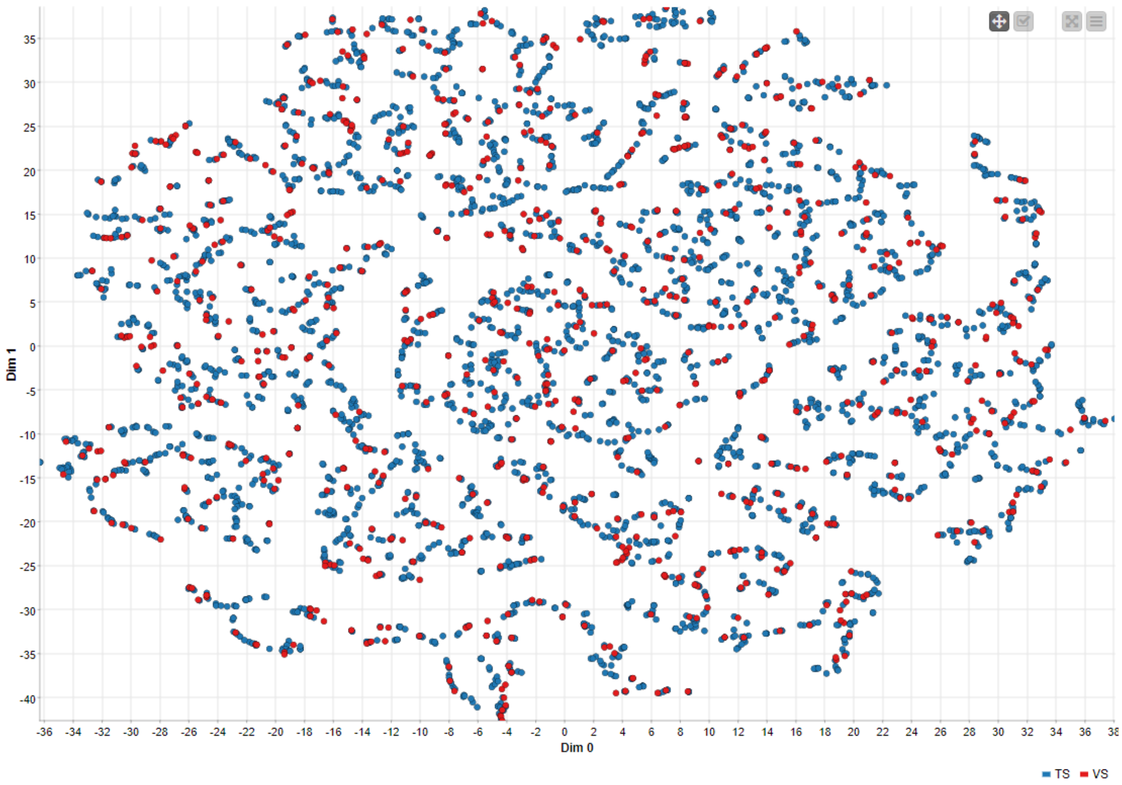

Figure S5: t-SNE plot visualizing training set and validation set. The dots in blue depict training set, and those in red depict validation. The homogeneous distribution of the chemicals (i.e no label specific clustering or pattern) indicate that the validation set is a representative of the training set data
