# Supplementary material for: Experimental and machine learning-based exploration of repurposed drugs reveals chemical features underlying phospholipidosis
Source: Patterns (N Y). 2026 Feb 6;7(4):101453. doi: 10.1016/j.patter.2025.101453 (PMC13083723; doi:10.1016/j.patter.2025.101453)
Supplement: Document S2. Article plus supplemental information [file mmc5.pdf]

Article

Patterns

Experimental and machine learning-based exploration of repurposed drugs reveals chemical features underlying phospholipidosis

Graphical abstract

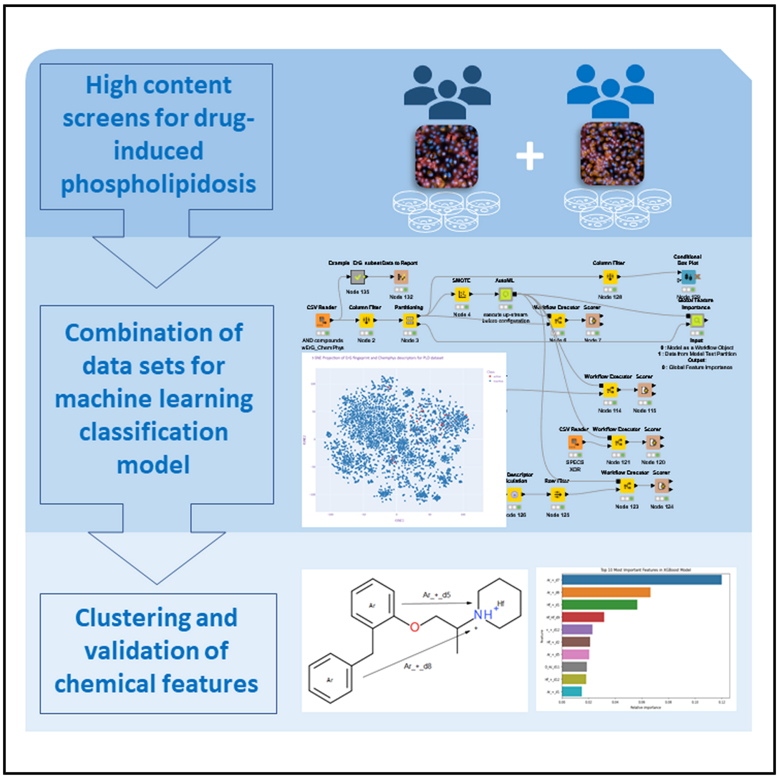

Highlights

- Repurposing collection dataset for drug-induced phospholipidosis in 2 cell lines
- A machine learning model using experimental phospholipidosis data across 2 cell lines
- Exploration of chemical features underlying phospholipidosis and cell susceptibilities
- Correlation of drug-induced phospholipidosis with phenotypic antiviral effect

Authors

Maria Kuzikov, Adelinn Kalman, Reagon Karki, ..., Yojana Gadiya, Philip Gribbon, Andrea Zaliani

Correspondence

maria.kuzikov@itmp.fraunhofer.de

In brief

Phospholipidosis (PLD) is a cellular adverse effect of excessive accumulation of phospholipids that can be triggered by many cationic amphiphilic drugs, therefore bringing challenges for drug discovery. Here, over 5,000 repurposed drugs are tested for PLD induction across multiple cell lines. A robust machine learning model was developed and validated to accurately predict PLD-inducing compounds, offering a powerful early-screening tool to accelerate drug discovery and minimize costly late-stage failures.

Kuzikov et al., 2026, Patterns 7, 101453
April 10, 2026 © 2025 The Author(s). Published by Elsevier Inc.
https://doi.org/10.1016/j.patter.2025.101453

Patterns

CellPress
OPEN ACCESS

Article

Experimental and machine learning-based exploration of repurposed drugs reveals chemical features underlying phospholipidosis

Maria Kuzikov,[1,6,*] Adelinn Kalman,[2] Reagon Karki,[1] Jeanette Reinshagen,[1] Johanna Huchting,[1] Kun Qian,[3,4] Hanna Axelsson,[3,4] Marianna Tampere,[2] Päivi Östling,[2] Brinton Seashore-Ludlow,[2,3,4] Yojana Gadiya,[1,5] Philip Gribbon,[1] and Andrea Zaliani[1]

[1]Fraunhofer Institute for Translational Medicine and Pharmacology (ITMP), Schnackenburgallee 114, 22525 Hamburg, Germany
[2]Department of Oncology-Pathology, Karolinska Institutet, Science for Life Laboratory, Solna, Sweden
[3]Department of Medical Biochemistry and Biophysics, Karolinska Institutet, Science for Life Laboratory, Solna, Sweden
[4]Chemical Biology Consortium Sweden, Science for Life Laboratory, Department of Medical Biochemistry and Biophysics, Karolinska Institutet, 171 77 Stockholm, Sweden
[5]Bonn-Aachen International Center for Information Technology (B-IT), University of Bonn, 53113 Bonn, Germany
[6]Lead contact
*Correspondence: maria.kuzikov@itmp.fraunhofer.de
https://doi.org/10.1016/j.patter.2025.101453

THE BIGGER PICTURE Phospholipidosis is a cellular condition characterized by the excessive accumulation of phospholipids within cells, and it can also be induced by medications, especially those known as cationic amphiphilic drugs. While this phenomenon is not always harmful in itself, it can interfere with how cells respond in laboratory experiments and complicate the interpretation of drug screening results, leading to potential delays or failures in the development of new therapies. As the pharmaceutical industry increasingly relies on high-throughput screening and machine learning to accelerate early-stage drug discovery, there is growing interest in identifying compounds that may cause phospholipidosis before they move too far through the development pipeline.

In response to this challenge, we at the EU-Horizon HLTH Remedi4ALL project compiled and analyzed one of the most comprehensive datasets to date—over 5,000 repurposed drugs tested across multiple cell lines—to better understand which compounds are likely to induce phospholipidosis. Using this dataset, we developed a machine learning model capable of predicting the risk of phospholipidosis based on a drug's chemical structure. By validating our approach across diverse experimental settings, our goal is to provide the broader scientific and pharmaceutical communities with a practical tool to flag problematic compounds early, ultimately helping bring safer, more effective treatments to patients faster.

SUMMARY

Phospholipidosis (PLD) is a cellular adverse effect caused by, among other things, cationic amphiphilic drugs. There is interest within pharma discovery to predict this phenomenon, as it can impact the outcome of phenotypic cellular screens and significantly delay drug development processes. The development of accurate and validated machine learning models for predicting drug-induced PLD across different cell lines and research centers could provide a valuable early application tool for the pharmaceutical industry, potentially accelerating drug discovery and reducing the risk of late-stage failures. We report here the assembly, curation, testing, and modeling of one of the largest datasets of repurposed drugs (5,000+) tested for PLD induction on different cell lines. A machine learning classification method was developed and validated to predict whether molecules are prone to induce PLD effects when applied in cell-based screens.

INTRODUCTION

Phospholipidosis (PLD) is a pathological storage disorder observed as an excessive accumulation of phospholipids in lysosomes.[1] PLD's involvement in various cellular processes makes it relevant to multiple therapeutic areas, including oncology, neurology, and immunology. The mechanism behind drug-induced PLD is not well understood; it is thought to involve

© 2025 The Author(s). Published by Elsevier Inc.
This is an open access article under the CC BY license (http://creativecommons.org/licenses/by/4.0/).

the inhibition of lysosomal phospholipase activity, an increase of lysosomal pH toward neutral, blockage of mannose-6-phosphate receptor (M6PR) leading to the inhibition of lysosomal hydrolase activities, and changes of lysosomal membrane composition.[2,3]

Special attention is paid when these conditions are induced by chemical entities. Cationic amphiphilic drugs, e.g., chloroquine or tamoxifen, were reported to induce PLD *in vitro* and *in vivo.*[4,5] Emerging evidence suggests that PLD may also impair virus infection and the replication cycle *in vitro.*[4] On the one hand, viruses remodel the host's cellular environment, including phospholipid metabolism, to create optimal conditions for their replication. On the other hand, phospholipids and their regulatory enzymes influence virus replication. For instance, the presence of phospholipids on the plasma membrane prevents the attachment of the respiratory syncytial virus (RSV), while cholesterol oxidation product 25-hydroxycholesterol (25HC) impairs hepatitis C virus infection by disrupting the formation of the virus-induced double-membrane vesicle network necessary for its replication.[6,7] However, as highlighted by Tunnimo et al. for SARS-CoV-2, the antiviral effects associated with drug-induced PLD have not been consistently replicated *in vivo*, leading to concerns that drug-induced PLD may be an artificially generated antiviral effect that may result in the generation of false positive (FP) results in cell-based antiviral drug screening.[4] These findings highlight the need for investigation into the chemical properties of compounds that lead to PLD induction in order to minimize FPs that might not have a real protein target as the basis of their action.

Studies on common chemical properties for PLD-inducing compounds have been reported in the literature since the 1970s, i.e., the presence of a tertiary amine group that becomes protonated and subsequently trapped inside negatively charged lysosomal vesicles or a hydrophobic ring structure with a hydrophilic side chain.[8–11] Several studies have now demonstrated applications of machine learning (ML) techniques for predicting compounds' propensity to induce PLD. One of the earliest efforts dates back to 2008, when Ivanciuc trained and compared over 20 different ML models using the Weka software to explore the structure-activity relationship (SAR) behind PLD.[12–14] The training set comprised structural descriptors for 201 compounds, 118 of which were publicly available, and the remaining ones were taken from Pfizer's internal database. From the comparison, Ivanciuc reported that a support vector machine (SVM) with a Gaussian radial basis function (RBF) kernel achieved the highest performance in a 10-fold cross-validation approach with an accuracy of 97% and a Matthews correlation coefficient (MCC) of 0.94. Several years later, Lowe et al. expanded the dataset using literature-derived data and developed ML models based on structural and circular fingerprints, as well as a combination of both.[15] They found that circular fingerprints alone led to better models, with random forest (RF) and SVM models showing similarly high performance. Their best models achieved over 90% accuracy in predicting PLD-inducing potential. Later, in 2012, Orogo et al. constructed a dataset of 743 compounds and investigated the effectiveness of commercially available QSAR software programs in PLD prediction.[16] Their commercial model, which integrated multiple ML approaches, achieved 86% accuracy in predicting PLD. In another study, Fusani et al. combined high-content screening data with *in silico* modeling to predict PLD risk.[17] Using a mix of public and proprietary data from AstraZeneca, they found that the SVM model outperformed the RF, achieving 86% accuracy on an external test set. In recent years, interest from both pharmaceutical companies and academia has driven further advances. Boehringer Ingelheim used their *in vitro* assay results to explore experimental and computational approaches for PLD-inducing compounds,[18] while academic researchers applied cell-imaging techniques.[19] Among these efforts, the AMALPHI (https://www.ba.ic.cnr.it/softwareic/amalphi/) ML platform developed by Lomuscio et al. stands out for its accessibility, as it openly provides the model for broader use.[20] Their best model achieved 88% accuracy and an MCC of 0.76 on the test set, expanding the utility of PLD prediction models beyond traditional user groups. Collectively, these studies have demonstrated two key insights: (1) understanding the relationship between compound structure and PLD can offer valuable clues about PLD-related toxicity and (2) larger, more comprehensive datasets enable the discovery of new findings and structural characteristics for compounds' PLD effects. As datasets continue to grow and ML techniques evolve, the ability to predict and mitigate PLD-related toxicity will likely improve, leading to safer drug development and more informed therapeutic strategies.

Our findings delve deeper into the risk of FPs associated with PLD in screening, particularly in the context of antiviral drug discovery, with implications seen during the recent SARS-CoV-2 pandemic. Finally, we make available the ML model, its source code, and training data in public repositories, ensuring openness and data sustainability for future research.

RESULTS

Identification of PLD-inducing compounds from drug-repurposing collection

To investigate PLD in an experimental setup, we used 5,228 compounds from the repurposing collection inspired by the Broad Repurposing Library (v.2017).[21] The compounds were screened at 10 μM in Vero-E6 and A549-ACE2 cells for induction of PLD after 24 h of treatment (Table S2). At first sight, our observations revealed that certain classes of compounds caused PLD across both cell lines, while others were exclusive to specific cell lines. This suggests a potential cell-line dependency in drug-induced PLD (Figure 1). In summary, according to the applied criteria described in building training and test sets, we observed 155 molecules inducing PLD in A549-ACE2 cells and 197 molecules inducing PLD in Vero-E6 cells. These numbers represent 2.7%–3.5% of the total drug-repurposing collection, respectively.

Next, we focused on generating a representative dataset showcasing the PLD effect. For this, we categorized compounds as "active" or "inactive" based on a PLD induction threshold of >52% for A549-ACE2 cells and >50% for Vero-E6 cells. These different thresholds enabled adjusting for experimental variability across institutes (Institute for Translational Medicine and Pharmacology [ITMP] and Karolinska Institute [KI]), due to the use of different positive controls (ITMP: amiodarone 10 μM, KI: tamoxifen at 10 μM) for PLD induction, which were used to normalize the data and cell lines. We apply high threshold values as we use our primary screen data, which means single-point

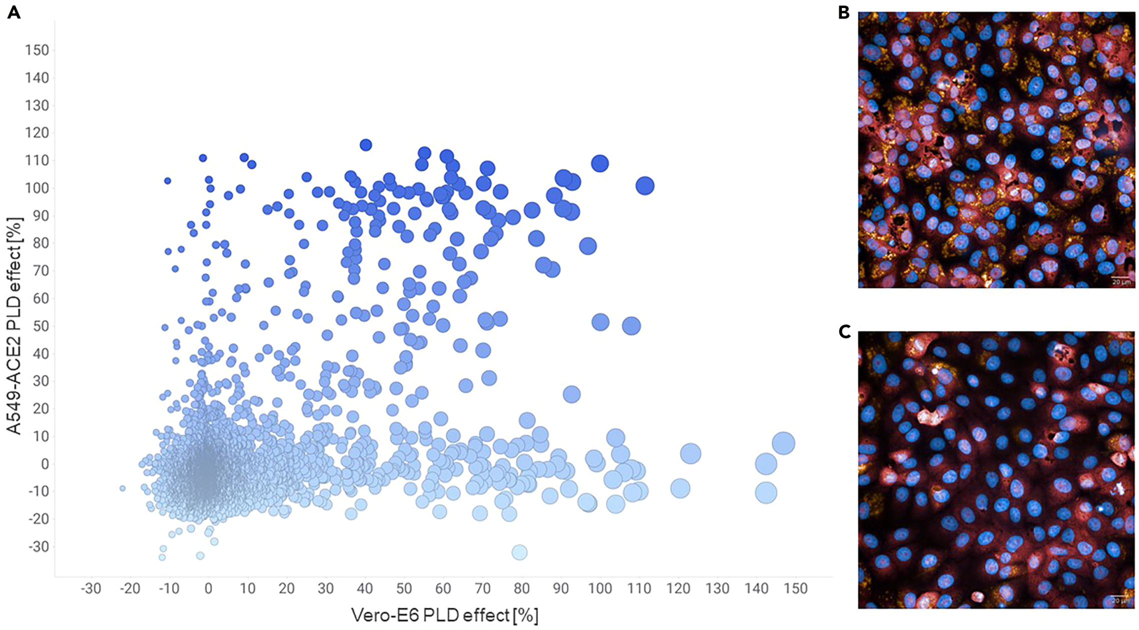

Figure 1. Correlation of PLD in Vero-E6 and A549-ACE2 cells

(A) Plot showing the correlation of the PLD induction screening results between Vero-E6 cells and A549-ACE2 cells. Data points are marked by color for the A549-ACE2 PLD effect and by dot size for the Vero-E6 PLD effect.

(B) PLD induction in Vero-E6 cells using 20 μM amiodarone (positive control for PLD induction).

(C) Vero-E6 cells incubated with 0.1 v/v % DMSO (negative control for PLD induction). Blue: nuclei stain, red: cytoplasm stain, and orange: PLD detection.

values for each compound at one concentration (10 μM), and we aim to train our model on strong PLD inducers across two different cell lines. Ultimately, from the 5,228 compounds tested, 4,258 compounds with consistent and comparable results across cell lines (i.e., active or inactive in both cell lines) were selected as the representative set and used for ML modeling (Figure 2). After selecting the dataset, we divided the 4,258 compounds into training and testing subsets using an 80:20 split ratio. The chemical structures in the dataset were converted into vector features (through fingerprints) to represent their characteristics, enabling their use as input for the ML model (see building training and test sets for more details). To ensure the validity of the testing dataset, we carefully analyzed its underlying chemical space prior to model training.

To assess the distribution and uniformity of the chemical space across the data (4,258 compounds) used in ML modeling, we employed a t-SNE (t-distributed stochastic neighbor embedding) plot, as shown in Figure 3. This visualization was critical in confirming that the chemical space modeled through the extended-reduced graph (ErG) fingerprinting method, combined with chemico-physical (ChemPhys) descriptors, was both homogeneous and representative of the broader dataset. Such inspection ensured that the dataset was free from significant biases, which could otherwise compromise the model's ability to generalize effectively.

Enhanced performance with boosted tree models

Our training approach involved evaluating six ML models (gradient boosted tree [GB], logistic regression [LR], neural network [NN], decision tree [DT], RF, and extreme gradient boosting [XGBoost]) using a 4-fold cross-validation strategy. This method allowed us to systematically assess model performance across these ML algorithms. The results revealed that algorithms based on boosted trees performed slightly better than the other models. However, the differences in accuracy were initially overestimated due to a bias in the dataset favoring inactive compounds. When additional performance metrics, such as false negatives (FNs) and FPs, as reported in Table 1, were considered, the superior performance of XGBoost became more evident. These metrics provided a clearer and more balanced evaluation of the model's ability to correctly identify active and inactive compounds. We, therefore, trained the final model with all 4,258 compounds with the XGBoost algorithm for exploring feature importance and performing prediction in an external library.

For an analysis of key compound features leading to PLD induction, we have decided first to use the most classical ChemPhys descriptors; in a second step, we applied pharmacophoric fingerprints (ErG), which are associated with substructural moieties. Whereas the ChemPys is a set of descriptors that describe classical circular structural ECFP4/6, which are strictly dependent on structural graphs, the ErG is a recapitulation of the distribution of important pharmacophoric atoms. An analysis of the top 10 most relevant descriptors identified by the model highlights two key observations (Figure S1). First, the ChemPhys descriptors appear to be less effective in modeling the problem than initially expected. While characteristics such as cell permeability or solubility, approximated by higher calculated topological polar surface area (TPSA) or logP values, might be anticipated to influence the PLD effect, they did not appear to

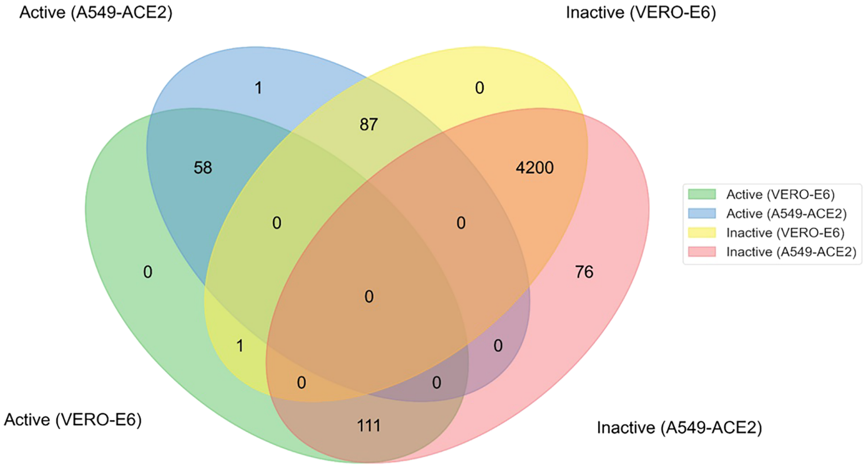

Figure 2. Overlap of the compounds across cell lines

In total, 4,200 compounds found to be inactive in both screens (Vero-E6 and A549-ACE2) were added to 58 compounds found to be active in both screens. The remaining compounds (1,192 compounds) comprise actives or inactives in one of the screens.

play a significant role in this context. Second, eight out of the ten most relevant features contained stable positively charged atoms at physiological pH, aligning with the known properties of cationic amphiphilic drugs, which are well documented as PLD inducers.[22] Additionally, specific molecular structural features, many of which have been validated in previous studies,[5] were instrumental in driving the model's accurate predictions. For example, several ErG bits describing the distance between aromatic centroids and a positive charge (e.g., Ar_+_d7, Ar_+_d6, Ar_+_d5, and Ar_+_d1) ranked first, second, seventh, and tenth, respectively, in the relevance list (Figure 4). Closely related to this finding is the identification of Hf_+_d1 (ranked third), Hf_+_d2 (ranked sixth), and Hf_+_d12 (ranked ninth) among the most relevant descriptors (Figure S1). The Hf_+_d1 structural feature corresponds to fragments where isopropyl (iPr), isobutyl (iBu), or lipophilic fatty chains are attached to a nitrogen atom that remains stably charged at physiological pH. This feature frequently includes piperidine-based tertiary or quaternary nitrogen structures. Interestingly, some previously unrecognized structural pharmacophore features also emerged as significant, particularly those representing long-distance interactions. For instance, Hf_Hf_d9 (ranked fifth), +_+_d12 (ranked sixth), and D_Ac_d11 (ranked eighth) have not been reported in prior studies. These descriptors suggest new avenues for understanding the molecular basis of the modeled phenomena and highlight the importance of considering unconventional structural features in future analyses. The real value differences for the top 10 features are reported in Figure S2 as boxplots,

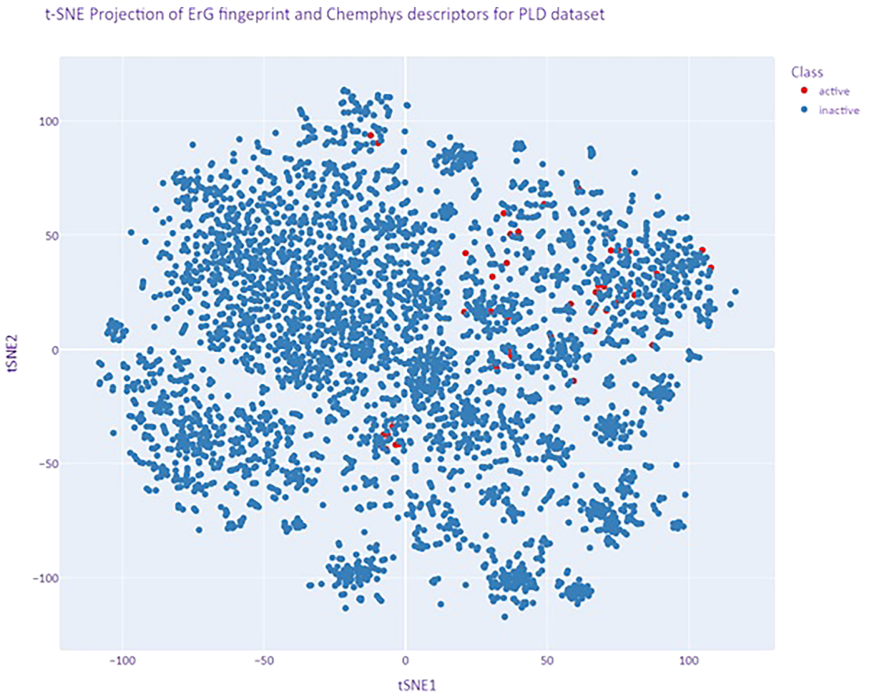

Figure 3. t-SNE plot of the Broad Repurposing Library

The dots in blue demonstrate PLD-inactive compounds, and those in red highlight PLD-active compounds. The homogeneous distribution of actives within the library is evident, as it does not show any particular clustering in the chemical space described by chemical descriptors.

CellPress
OPEN ACCESS

Table 1. Classification algorithm performances on the validation set (*n* = 852)

Algorithm	Test accuracy	AUC	F1 measure	Sensitivity	Specificity
Gradient boosted trees	0.9952	0.9998	**0.9963**	0.9970	0.9955
Logistic regression	0.9773	0.9913	0.9789	**1.0000**	0.9568
Neural network	0.9870	0.9964	0.9882	**1.0000**	**0.9761**
Decision trees	0.9818	0.9915	0.9830	0.9895	**0.9761**
Random forest	0.9588	0.9995	0.9614	**1.0000**	0.9196
XGBoost	**0.9955**	**0.9999**	0.9955	0.9955	0.9955

The highest values for each metric are bolded.

together with the relative statistical *p* values. Very few non-significant differences were found.

The final XGBoost model was used to predict actives/inactives in an external library. For this purpose, we chose the US Food and Drug Administration (FDA) collection of 774 drugs, referred to here as ENZO (https://www.enzo.com/basic-research/technology-platforms/small-molecule-chemistry/compound-libraries/). The collection included approved drugs, therefore reflecting the general features of the 5,228 compound-repurposing collection used in the primary screen. Of the entire collection, 13 compounds (1.7%) were predicted to induce PLD. Compounds labeled as active (P label > 0.5, with P label = probability value to be active with label = 1 against the inactive label = 0) were subsequently tested in Vero-E6 cells under the same experimental conditions used in the training set. To assess the model's transferability to cell lines not included in the training data but frequently used as cellular models, especially in phenotypic (antiviral) screens, additional tests were conducted using A549 and CaCo2 cells. Using the threshold criteria of 30% PLD induction at a concentration of 10 μM, 11 out of the 13 predicted compounds (84.6% confirmation rate) demonstrated PLD induction in Vero-E6 cells (Table 2). Similarly, 12 out of 13 compounds (92.3% confirmation rate) showed PLD activity in A549 cells. In CaCo2 cells, 8 out of the 13 compounds (66.7% confirmation rate) were confirmed to induce PLD. The cutoff criteria were adjusted to 30% due to the different potencies of amiodarone across the tested cell lines, which was used as a positive control for PLD induction and therefore for the normalization of results. The threshold of 30% represents a significant increase in the PLD effect in cells.

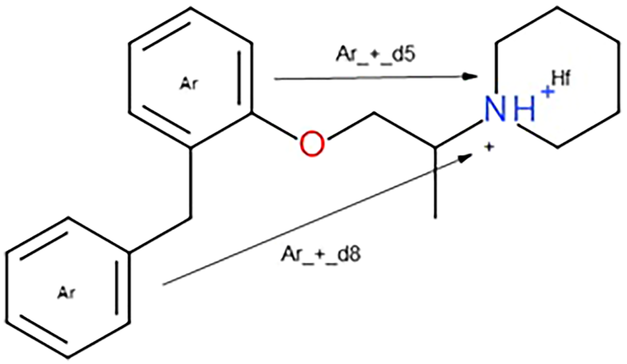

Figure 4. Scheme of benproperine, an antitussive compound with high PLD propensity, representing how the ErG fingerprint scheme coded the relevant distances

Correlation of drug-induced PLD with phenotypic screens for SARS-CoV-2 inhibition/antiviral activities

The interest in understanding PLD induction and its predictive modeling has recently grown, particularly due to observations that antiviral compound effects against SARS-CoV-2, often associated with PLD induction, have not been consistently replicated *in vivo*.[4] This raises the question of how much PLD might confound screening results, as seen in aggregation issues during SARS-CoV-2 drug screens or other antipathogenic assays.[23,24] In our recent work, we published several phenotypic anti-SARS-CoV-2 screening results, which are now available in public databases (CHEMBL4495565 for Vero-E6 cells and CHEMBL4303101 for CaCo2 cells).[25,26] Here, we use the data to investigate the drug-induced PLD in our phenotypic anti-SARS-CoV-2 screens. By using the same drug-repurposing collection, handling procedures, and material sources, we avoid discrepancies to make the datasets of the screens maximally comparable. Figure 5 illustrates the correlation between the inhibition of the cytopathic effect (CPE) caused by SARS-CoV-2 infection and drug-induced PLD observed in these phenotypic screens. For this drug-repurposing collection, no significant positive correlation was observed between PLD induction and CPE inhibition. When comparing assays across cell lines, Vero-E6 cells appeared more restrictive than CaCo2 cells in terms of identifying compounds with antiviral activity. On the other hand, we saw from the test of the ENZO library collection that Vero-E6 cells seem to show higher sensitivity to PLD compared to CaCo2 cells. By correlating PLD induction and CPE inhibition in Vero-E6 cells, 2 out of the 35 compounds with an inhibition of the CPE of >30% also exhibited a PLD induction of >50% (5.7% of the total hits). In CaCo2 cells, 68 out of 956 compounds (7.1% of the total hits) showed an inhibition of the CPE of >30% and a PLD induction of >50%. This observation suggests that although the total number of anti-CPE compounds seems to be very different, the percentage of compounds among anti-CPEs that also show PLD is quite similar. These findings highlight a potential confounding factor in anti-SARS-CoV-2 screening programs, as, although there is no direct positive correlation, drugs inducing PLD, alongside antiviral effects, may be falsely identified as hits.

The question of how much of the observed antiviral activity can be imputed to PLD induction is certainly legitimate. The

Table 2. Summary of experimental PLD assay results of the ENZO library on three different cell lines measured at a 10 μM in-assay concentration

Name	Structure	P (label = active)	P (label = inactive)	PLD effect % in Vero-E6	PLD effect % in A549	PLD effect % in CaCo2
Chlorpromazine	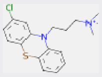	0.60	0.40	116.1	80.8	67.1
Fluoxetine	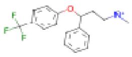	0.99	0.01	111.3	107.7	50.5
Trifluoperazine	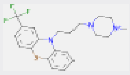	0.54	0.46	101.9	97.8	63.2
Duloxetine	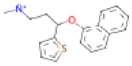	0.92	0.08	95.9	108.4	84.9
Promethazine	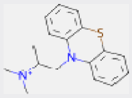	0.89	0.11	90.7	81.9	37.8
Fluphenazine	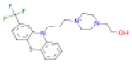	0.54	0.46	90.3	77.4	95.4
Azelastine	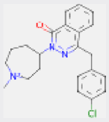	0.87	0.13	81.7	89.8	36.5
Clomipramine	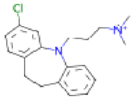	0.54	0.46	70.9	66.2	74.6
Thioridazine	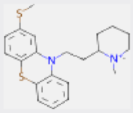	0.87	0.13	44.7	33.5	22.7
Cinacalcet	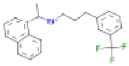	0.78	0.22	43.0	105.1	20.9
Clemastine	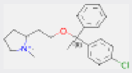	0.69	0.31	30.1	50.4	6.9
Atomoxetine	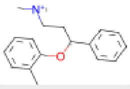	0.79	0.21	27.7	70.9	0.7
Naftifine	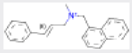	0.73	0.27	−1.6	7.5	−3.3

presented correlation is based on primary screening results for both PLD and anti-CPEs expressed as a percentage of the positive control measured at 10 μM. A deeper look into drug-induced PLD EC50s and anti-CPE EC50s can reveal a notion, which may improve the discrimination between PLD and anti-CPEs in some antiviral screening. From 68 dual active compounds in CaCo2 cells, we have selected compounds with strong CPE inhibition (>50%) and PLD induction (>50%), including the 2 dual active compounds in Vero-E6. These compounds were tested for dose response in Vero-E6 cells to determine the PLD EC50, and the results correlated with available anti-SARS-CoV-2 CPE EC50 data (Table S1). From the dataset, we identified 9 compounds for which both anti-CPE and PLD EC50 data were available: NNC-55-0396, lidoflazine, PD-161570, U-18666A,

CellPress
OPEN ACCESS

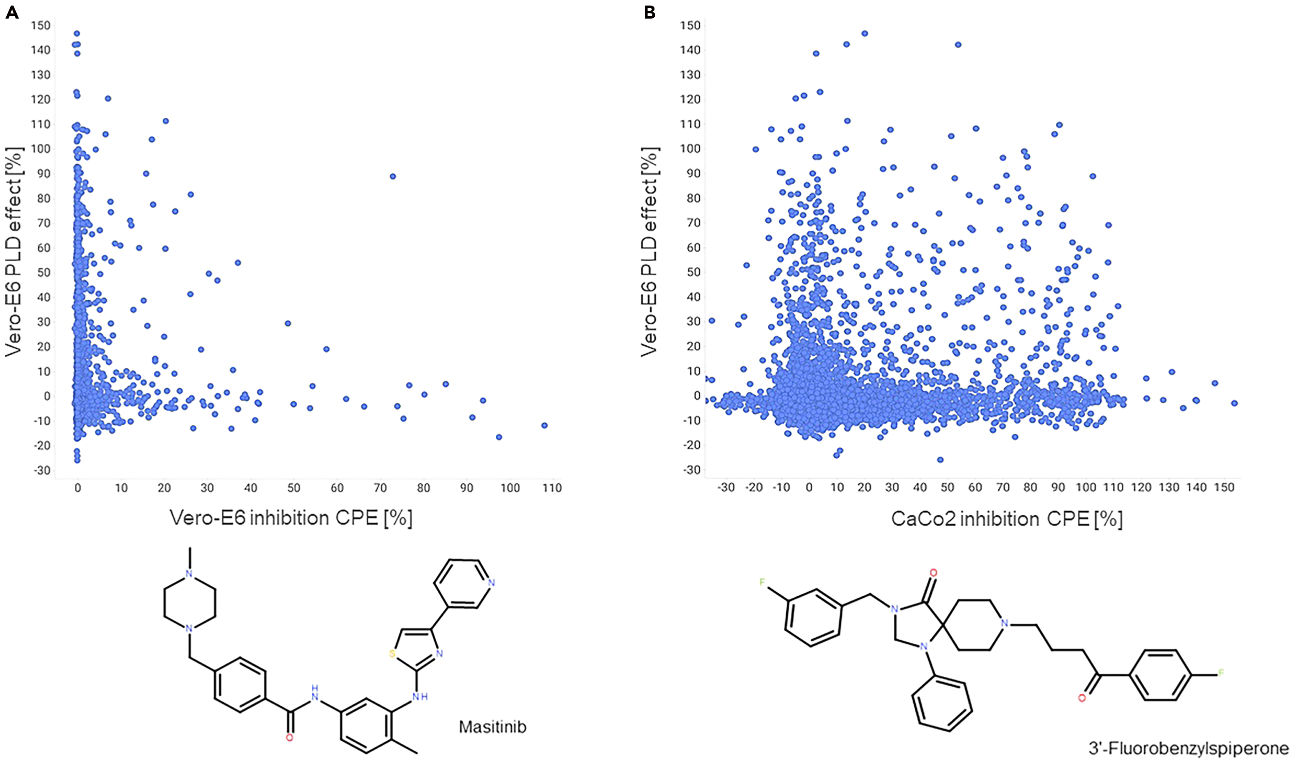

Figure 5. Correlation of PLD induction and SARS-CoV-2 CPE inhibition

(A) Scatterplot correlating PLD induction in Vero-E6 cells with CPE inhibition as reported in the CHEMBL4495565 CPE assay on Vero-E6 cells.

(B) Scatterplot correlating PLD induction in Vero-E6 cells with CPE inhibition in Caco2 cells as reported in the CHEMBL4303101 CPE assay. Bottom: two examples of active found compounds in the PLD induction assay and in the Vero-E6 and CaCo2 CPE assay: masitinib and 3′-fluorobenzylspiperone.

mefloquine hydrochloride, casin, amodiaquine dihydrochloride, 3′-fluorobenzyl spiperone, and masitinib (AB1010). We observed that for these compounds, the anti-CPE EC50 was higher than the calculated PLD EC50, meaning that the phenotypic antiviral effect was observed upon the onset of PLD induction. For 16 compounds, we also observed that the highest concentration of 20 μM resulted in the onset of a toxic effect, shown as negative PLD % effect values. Although the dataset represents a relatively low sampling size, the calculated EC50 revealed no drug-induced PLD activity at the low-nM level. Most compounds showed an EC50 between 1 and 3 μM, suggesting a potential activity range of this drug-repurposing collection.

DISCUSSION

PLD is a long-known storage disorder of accumulated phospholipids in lysosomes, with the first research results published in early 1948. Among the classical counter screenings used, PLD might play a secondary role when compared to P-glycoprotein efflux pump (PgP,) human ether-a-go-go related gene (hERG) potassium channel, or specific selectivity screening. However, in certain fields, such as antimicrobial and antiviral efforts, PLD plays an important role either in early infection (cell penetration) or late pathogen maturation mechanisms, including cell exit pathways. In some cases, we witnessed compounds with proven antimicrobial and antiviral activity, which, in reality, turned out to be interfering with host mechanisms of lipid assembly or trafficking. In recent years, the pharma industry has raised interest in testing and predicting this phenomenon, not least because the antiviral effects associated with drug-induced PLD in SARS-CoV-2 research have not been consistently replicated *in vivo*, but also due to the increasing focus of the FDA on the potential toxicity of drug-induced PLD. Drug-induced PLD is part of the FDA Adverse Event Reporting System (FAERS), with 60 reported severe cases of drug side effects in the last 5 years, among them one case of death.[27]

Therefore, we generated and analyzed the largest reported dataset of repurposed drugs, which includes 5,228 clinical compounds and drug candidates for PLD induction in two different cell lines. Our results revealed 58 compounds able to induce PLD in both cell lines at a concentration of 10 μM. Most of the compounds were preclinical candidates. Using the joint effort for annotation of the compounds in drug-repurposing libraries from the R4All consortia and ChEMBL v.34, we identified the corresponding targets for these compounds (Figure S3; Table S3).[28] We observed an accumulation of G-protein-coupled receptor (GPCR) active compounds, including, among others, histamine receptors H1, H3, and H4 and serotonin receptors HTR1A, HTR2A, and HTR2C. We also see compounds active on the neurotransmitter transporters, including serotonin, dopamine, and norepinephrine transporters (SLC6A4, SLC6A3, and SLC6A2) and several enzyme classes, such as kinases from the AKT family, protein methyl-transferases, and cytochrome P450 monooxygenases.

With a rigorous approach to merge different screening results by removing from the training dataset the molecules with inconsistent activity in different cell lines and addressing specificities of a repurposing collection, such as density in the chemical field or underrepresentation of the "actives class," we developed an ML classification method. The application of the model allowed us to successfully predict external repurposing collections such as ENZO, opening up a more general perspective for filtering library collections with certain PLD-inducing pharmacophoric chemical features. Among the chemical features collected by the model, the already known amphiphilic properties, involving stable charged nitrogen in diverse distances from hydrophobic groups, are certainly the most evident. However, within the top 10 most influential molecular features, there are important features described in the literature: for instance, the appearance of Hf_d1 and Hf_d2 showed evidently that the stable positive charge needs to be very close (one bond or two maximum) to lipophilic atoms. Such typical arrangements are di-isopropyl or di-butyl amino groups, which are known to be stably positively charged but easily water desolvatable. A totally novel and unprecedented molecular feature is D_D_12, i.e., a long-distance (12 bonds) pair of hydrogen-bond donors, which, however, is not present often in PLD-active molecules, as reported in the boxplots.

From experimental evaluation, we observed that, depending on the readout, 2.7%–3.5% of the total 5,228 compounds in the drug-repurposing collection are prone to inducing PLD. Also, the prediction for PLD-inducing compounds in the ENZO collection resulted in 1.7% being predicted as active. In contrast, our analysis of different library collections, such as LifeChem's (https://lifechemicals.com/screening-libraries/pre-plated-diversity-sets) 50k diversity set, using our prediction model resulted in 0.09% PLD-active hits. The results suggest a possible higher accumulation of different PLD-active compounds among the repurposed drugs and/or a more effective and precise filtering of the chemical space in commercial library design.

The application of Vero-E6 and A549-ACE2 in PLD screening already showed a difference in the sensitivity of cell lines for PLD. The observation was confirmed during the ENZO compound evaluation in Vero-E6, CaCo2, and A459-ACE2 cells. In addition, with EC50 determination, we observed that PLD can also be a toxic phenomenon when compound doses are increased by >20 μM. The effect of PLD itself, in most cases, was observed between a 1 and 10 μM compound concentration in assays; therefore, it most probably did not affect compound activities with in-assay concentrations below 1 μM. In our analysis of the impact of drug-induced PLD on the anti-SARS-CoV-2 compound effect, we saw no evident positive correlation between PLD induction and CPE inhibition in Vero-E6 and CaCo2 cells using the repurposing collection. It is not yet known whether the reported infection susceptibility is mechanistically linked to PLD susceptibility. However, for Vero-E6 and CaCo2 cells, the two susceptibilities appear to be correlated. For instance, when measuring SARS-CoV-2 infection susceptibility, Caco-2 cells were not highly susceptible to SARS-CoV-2 infection (25% infection rate), while Vero-E6 cells showed a 77% infection rate.[21] Also, during the ENZO compound evaluation, Vero-E6 cells showed higher PLD sensitivity (later confirmed for the whole ENZO library collection; data not shown).

What was not explored in this study, but certainly will have an impact on PLD induction, is the duration of exposure to the compound. The combination of cell line sensitivity, screening concentration, and exposure time will certainly impact how much PLD confounds the desired compound effect or will reveal PLD as a side effect in phenotypic screening for a compound.

The question lingering in the background, whether the induction of PLD can be leveraged in antiviral/antibacterial treatments or if it should be excluded from screening hit identification efforts, remains unanswered. Our study provides the scientific community with a comprehensive dataset for the analysis of PLD induction by repurposed drugs that, together with the ML model, allows a deeper exploration of chemical features of PLD induction and the impact on in-cell compound activity in the future.

METHODS

Experimental protocol for PLD

PLD induction was assessed at both the Fraunhofer ITMP and the KI using their respective drug-repurposing collections (https://compoundcenter.scilifelab.se/file/whatisavailable).[29] These collections were mainly developed with design features inspired by the Broad Repurposing collection and acquired in parallel from the same library provider (https://www.broadinstitute.org/drug-repurposing-hub).[30] We outline two protocols below, with ITMP conducting studies on Vero-E6 cells and KI on A549-ACE2 cells.

At ITMP, our experimental approach involves using Thermo's high-throughput screening (HTS)-optimized kit, which offers uniformity, reproducibility, and a narrow emission range suitable for multiplexing. To measure drug-induced PLD in Vero-E6 cells, we used the HCS LipidTOX Red Phospholipidosis Detection Reagent (Invitrogen, #H34351), which is non-toxic to cell growth and remains well retained after aldehyde fixation. In this setup, compounds (at 10 μM concentration) and controls (amiodarone: 10 μM concentration; DMSO: 0.1 v/v %) were added to clear-bottom, 384-well plates (PhenoPlate 384-well, black, optically clear flat bottom, tissue-culture treated, Revvity #6057302) using the Echo Liquid Handler (Labcyte). Vero-E6 cells were cultured in a complete medium: Dulbecco's modified Eagle medium (DMEM) supplemented with 10% FBS, 6 mM L-glutamine, and 1% penicillin/streptomycin. Cells were harvested at 80% confluence from a T175 cm^2 flask by washing once with 10 mL of room temperature (RT) PBS, followed by incubation with 5 mL of Trypsin 0.05%/EDTA 0.02% for 5 min. Cells were then resuspended in 10 mL of prewarmed culture medium. For plating, Vero-E6 cells were diluted to a concentration of 500,000 cells/mL in cell culture medium supplemented with a 1:1,000 dilution of HCS LipidTOX Red Phospholipidosis Detection Reagent (1,000×). A volume of 20 μL of this cell suspension was added to each well of the 384-well plate, and the cells were incubated for 24 h at 37°C in a 5% CO_2 environment. For fixation, 20 μL of 8% formaldehyde solution was added to each well and incubated for 30 min at RT. After incubation, the fixation buffer was removed, and the cells were washed with 50 μL/well of PBS. Cells were stained for 45 min with 20 μL/well of a solution containing CellMask Deep Red stain (1:3,000, Invitrogen #H32721) and Hoechst 33258 (1:10,000, Merck/Sigma-Aldrich #94403)

diluted in PBS. Following staining, the solution was removed, and the cells were washed three times with 50 μL/well of PBS. Image acquisition was performed using the automated Operetta Phenix High Content Screening system (Revvity). Image analysis was performed using Columbus 2.9 image analysis software (PerkinElmer) (Figure S4). Results were normalized against the positive control (amiodarone at 10 μM, set at 100% PLD induction) and the negative control (DMSO at 0.1% v/v, set at 0% PLD induction). Compounds with >50% PLD induction were classified as primary hits.

In a parallel effort at KI, assay-ready plates were prepared using the Echo (Labcyte) and stored in the freezer (−20°C) until use. Plates with pre-spotted compounds and controls were retrieved and thawed at RT for 30 min before seal removal. A549-ACE2 cells were cultured in a complete medium: DMEM/F-12 with GlutaMAX supplement (Thermo Fisher Scientific, 31331028), 10% FBS heat inactivated (Gibco, Thermo Fisher Scientific, 10500064), 1% penicillin/streptomycin (Cytiva, Nordic Biolabs, SV30010), and 1× NEAA (Cytiva, Nordic Biolabs, SH30238.01). Cells cultured in T175 flasks and reaching 70%–90% confluency were detached with trypsin and diluted to 1.3×10^5 cells/mL in media. The LipidTOX Green reagent (Thermo Fisher Scientific, #H34475) was diluted in the cell suspension to a final dilution of 1:1,000. This cell mixture was dispensed at 30 μL (4,000 cells/well) into the pre-spotted plates (PhenoPlate 384-well, black, optically clear flat bottom, tissue-culture treated, Revvity #6057302) using a Multidrop dispenser. Plates were stacked, covered with wet paper towels, and incubated in a plastic box inside a cell incubator. After 24 h, cells were fixed by adding 10 μL of 16% PFA (Thermo Fisher Scientific, #28908) to each well and incubated for 20 min at RT. The cells were washed three times with 80 μL of PBS using an Aquamax 4000 plate washer (Molecular Devices) and then incubated with 30 μL of a dye mix containing Hoechst 33342 (2 μg/mL, Thermo Fisher Scientific, #C10046) and CellMask Deep Red (1:20,000, Thermo Fisher Scientific, #C10046) in PBS for 1 h at 37°C. The plates were washed three additional times with 80 μL PBS and stored in 40 μL PBS at 4°C until imaging, after pre-warming for 10 min at RT. Images were captured using the ImageXpress Micro XLS Widefield High-Content Analysis System. Four fields of view were captured per well using a ×10 Plan Fluor 0.3 NA objective. Images were saved as 16-bit grayscale TIFF files, along with metadata. Image analysis was performed using CellProfiler.[31] For each well, cells were counted from nuclei staining, and cytoplasm was defined from CellMask staining. Within the cytoplasm, the number of phospholipid spots was counted from the LipidTOX Green staining. The average number of spots per cell per well was used to indicate induced PLD. Results were normalized against the positive control (tamoxifen at 10 μM, set at 100% PLD induction) and the negative control (DMSO at 0.1% v/v, set at 0% PLD induction).

Building training and test sets

The Broad Repurposing collection v.2017, present in both sites, comprising 5,632 compounds tested on both A549-ACE2 and Vero-E6 cells, formed the basis for our training set (https://repo-hub.broadinstitute.org/repurposing#download-data). We converted the compounds and their corresponding PLD induction results into a machine-readable, interpretable format. Due to variations in the PLD induction readouts between the two cell lines (see experimental protocol for PLD), we first filtered for compounds showing consistent results across both cell lines. Compounds were categorized as active or inactive based on a PLD induction threshold of >52% for A549-ACE2 cells and >50% for Vero-E6 cells, adjusting for experimental variability across institutes and cell lines. Ultimately, 4,258 compounds with consistent results across both cell lines were selected for ML modeling. Their structural information was first processed by desalting and calculating their dominant charge states and then was converted from SMILES structural representations to 2D fingerprint vectors to serve as ML model inputs. Using the RDKit library (https://www.rdkit.org/), we encoded the compounds into ErG[32] and 28 classical ChemPhys descriptors. The ErG fingerprint consists of 315 bits summarizing the pharmacophoric profile of each compound, while the ChemPhys fingerprint consists of 28 bits that represent the basic molecular properties of a compound, including TPSA, molecular weight (MW), logP, and other structural count-based descriptors. The selection of these fingerprints was driven by their interpretability and transparency, enhancing our understanding of the mechanics behind the ML model prediction. For model training, these fingerprints, combined with the PLD induction labels (active/inactive), were used as an input matrix to predict compound activity. The model training was performed with 3,406 compounds (i.e., 80% of PLD data), which were subjected to a 4-fold cross-validation strategy with an 80:20 split in the training and test sets. Afterward, the model assessment was done with a previously created subset of 852 compounds (i.e., 20% of PLD data), which were data unseen by the models.

In order to depict the closeness of the validation set with the training set, we generated a t-SNE plot using the above-mentioned ChemPhys and ErG fingerprints (Figure S5). It is evident from the plot that there are no specific patterns between these sets, which clearly suggests that the validation set is a representative of the training set chemical space. To further substantiate this observation, we performed a pairwise similarity analysis between all chemicals in the validation set against the training set. The analysis showed that 622 out of the 852 chemicals in the validation set have a Tanimoto similarity greater than 0.7 with at least one chemical from the training set. The full results of the analysis are provided in Table S4.

We then examined the distribution of PLD induction labels (active/inactive) within the training subset and observed a strong imbalance, with active compounds representing less than 2% and inactive compounds over 98%. To address this imbalance, we applied the synthetic minority over-sampling technique (SMOTE) method to generate synthetic samples, achieving an even distribution between the two labels.[33] The influence SMOTE had on the better performance of the model was also evaluated by comparing pre-SMOTE and post-SMOTE scenarios, where the latter yielded improved scores for accuracy, precision, and recall (see https://github.com/Fraunhofer-ITMP/PLD).

Training ML models

To identify the optimal ML model for our application, we began by training six classical ML algorithms using the AutoML node in KNIME (https://hub.knime.com/s/33fQGaQzuZByy6hE)

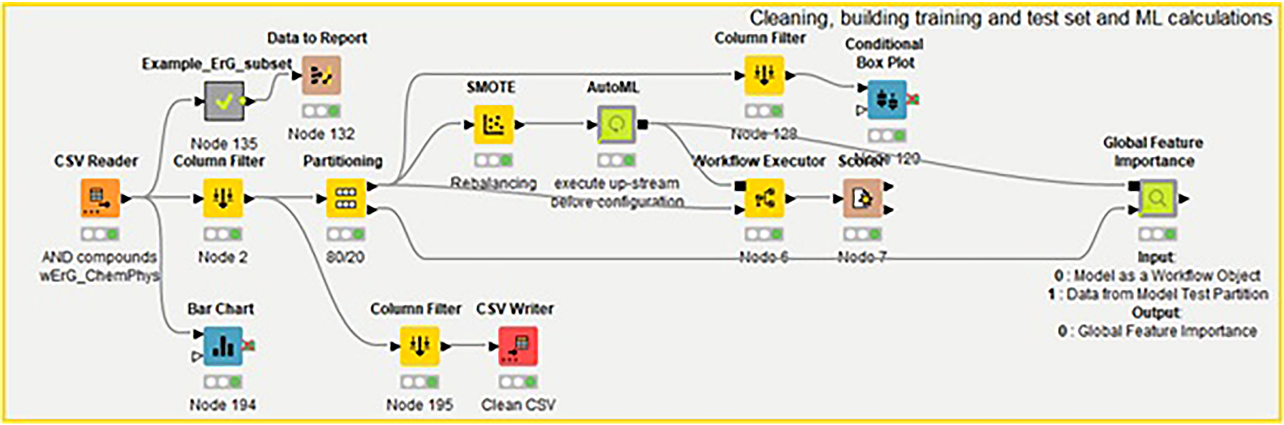

Figure 6. KNIME workflow used for ML development

This workflow begins with a CSV Reader node to load the training and testing datasets, which include ErG fingerprints and physicochemical descriptors. The central AutoML node then trains a selection of models, and for the top-performing models, feature importance is extracted using the global feature importance node.

(Figure 6). These algorithms included LR, a perceptron-based NN, GBs, DTs, RF, and XGBoost. The SMOTE-balanced training subset served as the input for this group of models. To ensure the robustness of the trained model, we performed 4-fold cross-validation, leaving out 20% of the data during each trial. The AutoML approach enabled us to identify the best-performing model from this cohort. In addition to the KNIME computational approach, we implemented a Python-based version to train and optimize the KNIME best-performing model (i.e., XGBoost classifier). For XGBoost hyperparameter optimization, we leveraged a refined GridSearch strategy provided by the scikit-learn library. The parameters used in the grid hyperparameter strategy include “n_estimators” at 700, “learning rate” at 0.025 and 0.05, “max depth” at 8 and 10, “gamma” at 0 and 1, “colsample bytree” at 0.5, and “min_child_weight” with values 2, 3, and 5.

In addition to model training and optimization, we conducted a feature importance analysis to identify the key chemo-physical properties that the models used to distinguish between active and inactive compounds in PLD induction. This was achieved using the global feature importance node in KNIME and the feature importance function within the XGBoost Python package (https://xgboost.readthedocs.io/en/stable/index.html). To assess model performance, we employed confusion matrices and Cohen’s kappa score, allowing us to evaluate and compare model accuracy and reliability. Alongside these metrics, we also report the F1 measure, sensitivity, and specificity as calculated below:

$$\text{F1 measure} = \frac{\text{true positive}}{\text{true positive}+\frac{\text{false postive}+\text{false negative}}{2}},$$

$$\text{sensitivity} = \frac{\text{true positive}}{\text{true positive}+\text{false negative}}, \text{and}$$

$$\text{specificity} = \frac{\text{true negative}}{\text{true negative}+\text{false positive}}.$$

RESOURCE AVAILABILITY

Lead contact

Requests for further information and resources should be directed to and will be fulfilled by the lead contact, Dr. Maria Kuzikov (maria.kuzikov@itmp.fraunhofer.de).

Materials availability

Both experimental protocols are outlined in detail in a standard operating procedure (SOP) recipe on Zenodo.[34] We will upload the PLD experimental results data in ChEMBL v.36 (expected to be released next year, 2026); in the meantime, the screening data used in this manuscript are attached as supplemental information and have been uploaded to Zenodo.[35]

Data and code availability

The source code and data files used in this manuscript can be found on resource-specific repositories: code on GitHub (https://github.com/Fraunhofer-ITMP/PLD), KNIME Hub (https://hub.knime.com/s/m6rnKt_4iYtDI1yt), and Zenodo.[36] The models have also been deployed on a public interface on the SERVE (https://serve.scilifelab.se/collections/remedi4all/) instance for community use.

Cellular imaging data will be uploaded to the Image Data Repository (IDR). Currently, the images are uploaded to BioImage Archive DOI 10.6019/S-BIAD2282 (S-BIAD2282 < Studies < BioStudies < EMBL-EBI).

ACKNOWLEDGMENTS

We would like to thank the authors of the resources used in our work for making their datasets available to the scientific community. We thank the Oscar Fernandez-Capetillo Laboratory, Karolinska Institutet, SciLifeLab, for generously providing A549-ACE2 cells. We would like to acknowledge Leonie Von Berlin for their feedback in improving the manuscript. We thank Jens Wendt (NFDI4BIOIMAGE, DFG project ID 501864659) for help with metadata annotation and upload to the BioImage Archive repository. We would also like to thank the anonymous reviewers for their comments and suggestions for improving the readability of the manuscript. This work and the authors were primarily funded by EU-Horizon HLTH Remedi4ALL (EU 101057442).

AUTHOR CONTRIBUTIONS

Conceptualization, M.K., Y.G., A.Z., J.R., and J.H.; investigation, M.K., A.K., K.Q., H.A., M.T., P.Ö., and B.S.-L.; data curation, M.K., A.Z., and J.R.; writing – original draft, M.K., Y.G., A.Z., J.R., and J.H.; writing – review & editing, R.K.,

J.H., P.G., and B.S.-L.; visualization, M.K., Y.G., R.K., and A.Z.; validation, M.K.; methodology, Y.G., R.K., A.Z., J.R., J.H., A.K., K.Q., H.A., M.T., P.Ö., and B.S.-L.; supervision, A.Z.; formal analysis, A.K., K.Q., H.A., M.T., P.Ö., and B.S.-L.;

DECLARATION OF INTERESTS

The authors declare no competing interests.

SUPPLEMENTAL INFORMATION

Supplemental information can be found online at https://doi.org/10.1016/j.patter.2025.101453.

Received: June 18, 2025
Revised: September 22, 2025
Accepted: November 20, 2025
Published: February 6, 2026

REFERENCES

1. Ceccarelli, M., Germani, R., Massari, S., Petit, C., Nurisso, A., Wolfender, J.L., and Goracci, L. (2015). Phospholipidosis effect of drugs by adsorption into lipid monolayers. Colloids Surf. B Biointerfaces *136*, 175–184.
2. Ikeda, K., Hirayama, M., Hirota, Y., Asa, E., Seki, J., and Tanaka, Y. (2008). Drug-induced phospholipidosis is caused by blockade of mannose 6-phosphate receptor-mediated targeting of lysosomal enzymes. Biochem. Biophys. Res. Commun. *377*, 268–274.
3. Abe, A., Hiraoka, M., and Shayman, J.A. (2007). A role for lysosomal phospholipase A2 in drug induced phospholipidosis. Drug Metab. Lett. *1*, 49–53.
4. Tummino, T.A., Rezelj, V.V., Fischer, B., Fischer, A., O'Meara, M.J., Monel, B., Vallet, T., White, K.M., Zhang, Z., Alon, A., et al. (2021). Drug-induced phospholipidosis confounds drug repurposing for SARS-CoV-2. Science *373*, 541–547.
5. Nonoyama, T., and Fukuda, R. (2008). Drug-induced Phospholipidosis -Pathological Aspects and Its Prediction. J. Toxicol. Pathol. *21*, 9–24.
6. Rattay, S., Hufbauer, M., Hoboth, P., Sztacho, M., and Akgül, B. (2023). Viruses and phospholipids: Friends and foes during infection. J. Med. Virol. *95*, e28658.
7. Blanchard, E., and Roingeard, P. (2018). The Hepatitis C Virus-Induced Membranous Web in Liver Tissue. Cells *7*, 191.
8. Halliwell, W.H. (1997). Cationic amphiphilic drug-induced phospholipidosis. Toxicol. Pathol. *25*, 53–60.
9. Breiden, B., and Sandhoff, K. (2021). Corrigendum to: Emerging mechanisms of drug-induced phospholipidosis. Biol. Chem. *403*, 251.
10. Shikata, T., Kanetaka, T., Endo, Y., and Nagashima, K. (1972). Drug-induced generalized phospholipidosis. Acta Pathol. Jpn. *22*, 517–531.
11. Yamamoto, A., Adachi, S., Kitani, T., Shinji, Y., and Seki, K. (1971). Drug-induced lipidosis in human cases and in animal experiments. Accumulation of an acidic glycerophospholipid. J. Biochem. *69*, 613–615.
12. Pelletier, D.J., Gehlhaar, D., Tilloy-Ellul, A., Johnson, T.O., and Greene, N. (2007). Evaluation of a published in silico model and construction of a novel Bayesian model for predicting phospholipidosis inducing potential. J. Chem. Inf. Model. *47*, 1196–1205.
13. Ivanciuc, O. (2008). Weka machine learning for predicting the phospholipidosis inducing potential. Curr. Top. Med. Chem. *8*, 1691–1709.
14. Kruhlak, N.L., Choi, S.S., Contrera, J.F., Weaver, J.L., Willard, J.M., Hastings, K.L., and Sancilio, L.F. (2008). Development of a phospholipidosis database and predictive quantitative structure-activity relationship (QSAR) models. Toxicol. Mech. Methods *18*, 217–227.
15. Lowe, R., Glen, R.C., and Mitchell, J.B.O. (2010). Predicting phospholipidosis using machine learning. Mol. Pharm. *7*, 1708–1714.
16. Orogo, A.M., Choi, S.S., Minnier, B.L., and Kruhlak, N.L. (2013). Notification: Construction and Consensus Performance of (Q)SAR Models for Predicting Phospholipidosis Using a Dataset of 743 Compounds. Mol. Inform. *32*, 121.
17. Fusani, L., Brown, M., Chen, H., Ahlberg, E., and Noeske, T. (2017). Predicting the Risk of Phospholipidosis with in Silico Models and an Image-Based in Vitro Screen. Mol. Pharm. *14*, 4346–4352.
18. Schieferdecker, S., Eberlein, A., Vock, E., and Beilmann, M. (2022). Development of an in silico consensus model for the prediction of the phospholipigenic potential of small molecules. Comput. Toxicol. *22*, 100226.
19. Hu, H., Tjaden, A., Knapp, S., Antolin, A.A., and Müller, S. (2023). A machine learning and live-cell imaging tool kit uncovers small molecules induced phospholipidosis. Cell Chem. Biol. *30*, 1634–1651.e6.
20. Lomuscio, M.C., Abate, C., Alberga, D., Laghezza, A., Corriero, N., Colabufo, N.A., Saviano, M., Delre, P., and Mangiatordi, G.F. (2024). AMALPHI: A Machine Learning Platform for Predicting Drug-Induced PhosphoLIpidosis. Mol. Pharm. *21*, 864–872.
21. Bennett, R.P., Postnikova, E.N., Eaton, B.P., Cai, Y., Yu, S., Smith, C.O., Liang, J., Zhou, H., Kocher, G.A., Murphy, M.J., et al. (2022). Sangivamycin is highly effective against SARS-CoV-2 in vitro and has favorable drug properties. JCI Insight *7*, e153165.
22. Lowe, R., Mussa, H.Y., Nigsch, F., Glen, R.C., and Mitchell, J.B. (2012). Predicting the mechanism of phospholipidosis. J. Cheminform. *4*, 2.
23. Glenn, I.S., Hall, L.N., Khalid, M.M., Ott, M., and Shoichet, B.K. (2023). Colloidal aggregation confounds cell-based Covid-19 antiviral screens. Preprint at bioRxiv. https://doi.org/10.1101/2023.10.27.564435.
24. Kumar, S., Li, J., Park, J., Hart, S.K., Song, N.L., Burrow, D.T., Bean, N.L., Jacobs, N.C., Coler-Reilly, A., Onyshchenko Pendergrass, A., et al. (2020). Sphingolipid biosynthesis inhibition as s host Sstrategy against diverse pathogens.. Preprint at bioRxiv. https://doi.org/10.1101/2020.04.10.035683.
25. Zaliani, A., Vangeel, L., Reinshagen, J., Iaconis, D., Kuzikov, M., Keminer, O., Wolf, M., Ellinger, B., Esposito, F., Corona, A., et al. (2022). Cytopathic SARS-CoV-2 screening on VERO-E6 cells in a large-scale repurposing effort. Sci. Data *9*, 405.
26. Ellinger, B., Bojkova, D., Zaliani, A., Cinatl, J., Claussen, C., Westhaus, S., Keminer, O., Reinshagen, J., Kuzikov, M., Wolf, M., et al. (2021). A SARS-CoV-2 cytopathicity dataset generated by high-content screening of a large drug repurposing collection. Sci. Data *8*, 70.
27. Potter, E., Reyes, M., Naples, J., and Dal Pan, G. (2025). FDA Adverse Event Reporting System (FAERS) Essentials: A Guide to Understanding, Applying, and Interpreting Adverse Event Data Reported to FAERS. Clin. Pharmacol. Ther. *118*, 567–582.
28. Reinshagen, J., Seashore-Ludlow, B., Gadiya, Y., Gustavsson, A.-L., Tanoli, Z., Aittokallio, T., Huchting, J., Jenmalm-Jensen, A., Gribbon, P., Zaliani, A., and Ballante, F. (2025). From library to landscape: integrative annotation workflows for compound libraries in drug repurposing. Database, baaf081.
29. Kuzikov, M., Costanzi, E., Reinshagen, J., Esposito, F., Vangeel, L., Wolf, M., Ellinger, B., Claussen, C., Geisslinger, G., Corona, A., et al. (2021). Identification of Inhibitors of SARS-CoV-2 3CL-Pro Enzymatic Activity Using a Small Molecule in Vitro Repurposing Screen. ACS Pharmacol. Transl. Sci. *4*, 1096–1110.
30. Corsello, S.M., Bittker, J.A., Liu, Z., Gould, J., McCarren, P., Hirschman, J.E., Johnston, S.E., Vrcic, A., Wong, B., Khan, M., et al. (2017). The Drug Repurposing Hub: a next-generation drug library and information resource. Nat. Med. *23*, 405–408.
31. Stirling, D.R., Swain-Bowden, M.J., Lucas, A.M., Carpenter, A.E., Cimini, B.A., and Goodman, A. (2021). CellProfiler 4: improvements in speed, utility and usability. BMC Bioinf. *22*, 433.
32. Stiefl, N., Watson, I.A., Baumann, K., and Zaliani, A. (2006). ErG: 2D pharmacophore descriptions for scaffold hopping. J. Chem. Inf. Model. *46*, 208–220.

33. Chawla, N.V., Bowyer, K.W., Hall, L.O., and Kegelmeyer, W.P. (2002). SMOTE: Synthetic Minority Over-sampling Technique. J. Artif. Intell. Res. *16*, 321–357.

34. Kuzikov, M. (2024). REMEDi4ALL - Standard Operating Procedure - Detection of Drug-Induced-Phospholipidosis (Version 1). Zenodo. https://doi.org/10.5281/zenodo.13880870.

35. Kuzikov, M., Karki, R., and Seashore-Ludlow, B. (2025). PLD data for manuscript “Interdisciplinary Study on Drug-Induced-Phospholipidosis of Repurposing Libraries through Machine Learning and Experimental Evaluation in Different Cell Lines”. Zenodo. https://doi.org/10.5281/zenodo.17200736.

36. Kuzikov, M. (2025). Source code and data repository for manuscript titled “Interdisciplinary Study on Drug-Induced-Phospholipidosis of Repurposing Libraries through Machine Learning and Experimental Evaluation in Different Cell Lines”. Zenodo. https://doi.org/10.5281/zenodo.15013138.

Patterns, Volume *7*

Supplemental information

Experimental and machine learning-based exploration of repurposed drugs reveals chemical features underlying phospholipidosis

Maria Kuzikov, Adelinn Kalman, Reagon Karki, Jeanette Reinshagen, Johanna Huchting, Kun Qian, Hanna Axelsson, Marianna Tampere, Päivi Östling, Brinton Seashore-Ludlow, Yojana Gadiya, Philip Gribbon, and Andrea Zaliani

Supplementary material

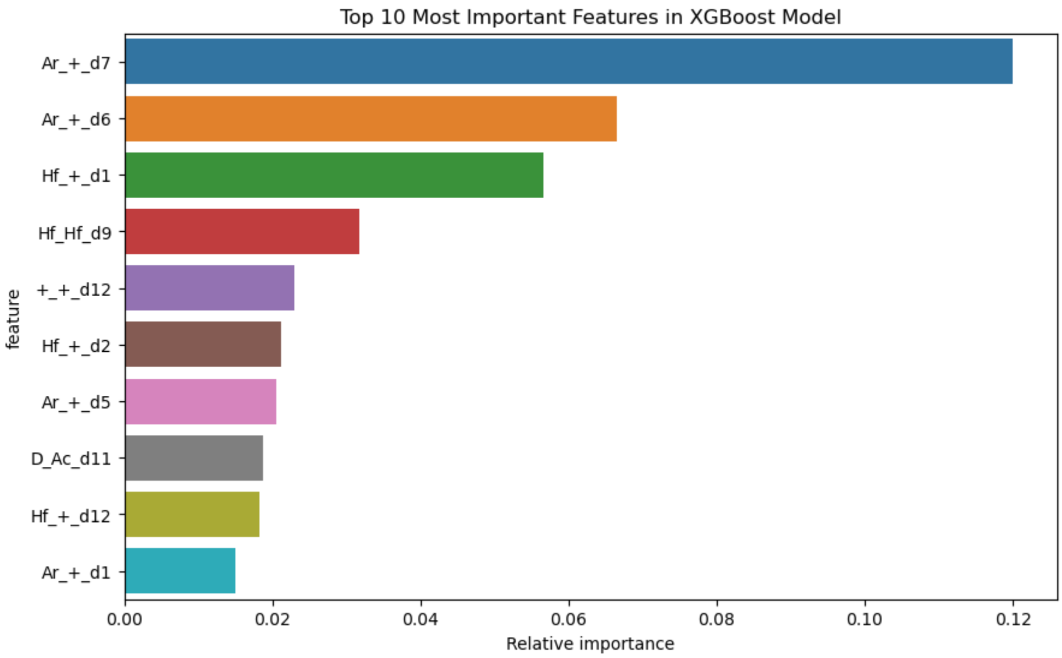

Figure S1: Bar chart reporting the top 10 most influential descriptors for the XGBoost model. The top features shown in this figure are the ErG annotations. In this notation, for instance, a descriptor like "Ar_+_d7" means that bits are set to 1 when an aromatic centroid lies seven bonds distant from a positive charge. Hf identifies a hydrophobic group (a collection of at least three carbon atoms), while "D" and "Ac" identify hydrogen-bond donors and acceptors, respectively.

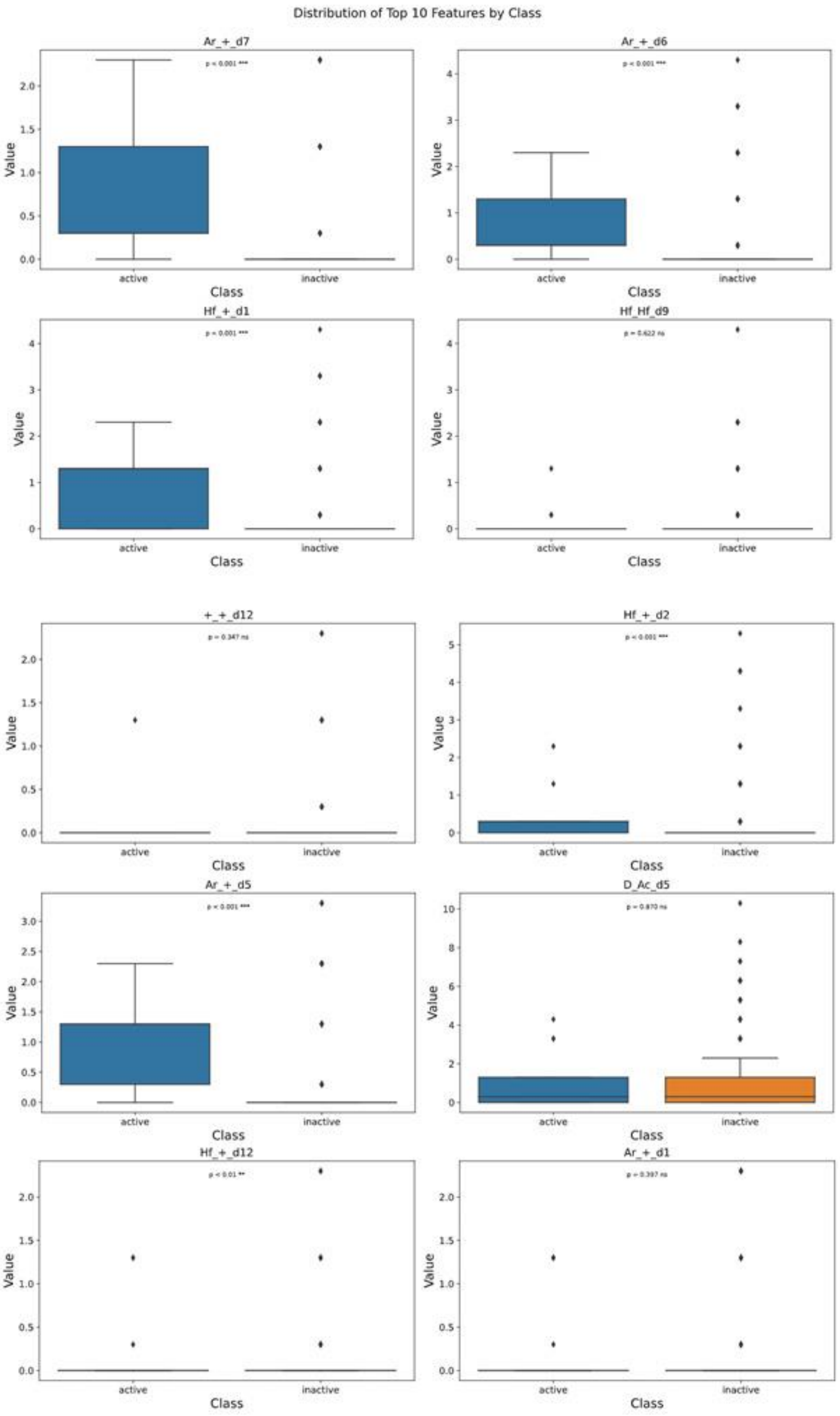

Figure S2: Distribution of the top10 most influential ErG features across the two classes with enhanced box plots. To understand the distribution across the two classes namely Active and Inactive in the original dataset. In almost all features chosen by the XGBoost model, Actives show higher values than Inactives. Significance p-values are given.

Table S1. PLD EC_{50} determination of anti-SARS-CoV-2 CPE active compounds, correlation to available CPE EC_{50}. PLD effect is normalized to amiodarone PLD induction effect at 10 µM, set to 100 %, DMSO is used as a negative control, set to 0 % PLD induction effect. Anti-SARS-CoV-2 CPE Data points are measured at 20 µM for infection in CaCo2 cells and at 10 µM for infection in Vero—E6 cells. Data points represent triplicate measurements. Empty fields: no data available

Effect in %								
Name	20 µM	10 µM	5 µM	EC50 µM	Vero-E6 CPE inhibition %	Vero-E6 EC_{50} µM	CaCo2 CPE inhibition %	CaCo2 EC_{50} µM
DDR1-IN-1	21.77	75.83	55.73	>20	-0.37		77.58	
TAK-960	-40.84	151.30	132.37	1.47	2.67		91.64	
SB-216641	-42.60	104.74	93.22	1.19	-0.22		57.65	
Lylamine	-41.01	133.55	104.48	>20	-0.36		76.43	
SB-743921	-43.61	223.62	171.82	2.49	-0.36		78.89	
DR-4485	-16.80	201.85	94.34	>20	-0.26		82.83	
Dexniguldipine	127.40	114.68	83.07	2.48	-0.15		97.57	
Cobimetinib	133.45	134.56	112.38	1.34	-0.31		61.58	
Lomitapide	-28.65	169.31	82.84	>20	-0.39		77.37	
Afatinib	146.21	248.65	128.55	>20	-0.39		60.16	
KHK-IN-1	toxic	toxic	toxic	toxic	toxic	toxic	toxic	toxic
NNC-55-0396	-43.08	154.81	87.06	6.28	0	8.21	58.09	
GR-127935	-40.19	161.77	136.46	1.12	-0.76		53.73	
Ceritinib	137.34	116.10	91.93	2.18	-0.35		59.98	
TAS-103	121.21	172.31	135.39	2.29	-0.08		92.56	
lidoflazine	129.06	80.16	65.28	5.51	0.5		95.92	17.39
tetrindole	-33.00	133.70	133.51	9.59	-0.34		75.19	
PD-161570	36.18	118.17	163.59	0.63	12.35	144.52	108.10	
U-18666A	112.55	98.22	84.91	1.77	9.72	1.15	77.44	
JHW-007	86.69	112.39	115.34	0.71	-0.32		93.14	
casin	134.63	193.97	116.35	3.31	-0.24	14.15	71.19	
chlorprothixene hydrochloride	191.97	112.70	124.38	2.36	0.27		55.65	
mefloquine hydrochloride	174.50	187.70	102.25	1.48	-0.32		93.28	14.15
NNC-05-2090	118.98	178.14	128.41	4.91	-0.32		71.83	
halofantrine HCl	113.55	86.98	79.75	1.66	0.17		91.38	
quinacrine hydrochloride	-13.21	141.12	145.20	1.55	-0.23		90.28	
amodiaquine dihydrochloride	186.68	101.86	88.75	>20	20.16	21.51	78.80	
norfluoxetine	197.87	111.51	92.13	1.96	-0.11		94.99	
metergoline	102.77	73.02	61.00	>20	0.05		54.65	
SNAP-5089	24.87	78.37	66.55	2.01	-0.12		72.63	
BiBX-1382	-0.64	75.46	85.75	0.61	0.28		79.29	
BIBU-1361	-42.53	180.15	160.51	0.63	-0.24		78.51	
tecalcet	101.68	74.29	59.63	2.6	-0.13		78.10	
tafenoquine	toxic	toxic	toxic	toxic	toxic	toxic	toxic	toxic
loperamide hydrochloride	86.14	75.44	55.03	2.74	5.03		58.93	

3'-fluorobenzylspiperone	8.92	41.30	61.76	10.3	72.81	7.66	102.38	
SR-33805 oxalate	137.08	86.07	54.44	9.26	0.02		67.72	
entrectinib (RXDX-101)	toxic	toxic	toxic	toxic	toxic	toxic	toxic	toxic
mibefradil	2.41	80.50	62.04	2.45	-0.26		52.45	
RG7112 (RO50455337)	88.45	141.41	109.12	2.56	6.32		88.52	
Vandetanib (ZD6474)	-32.66	136.39	128.97	1.05	0.29		51.24	
CID-2011756	55.98	31.32	27.66	>20	0.15		96.96	
ispinesib (SB-715992)	-43.20	205.19	206.65	1.81	-0.29		91.86	
AMG-073 HCl (cinacalcet hydrochloride)	252.97	79.48	70.80	18.58	-0.15		51.60	
masitinib (AB1010)	53.29	45.24	43.15	2.57	37	11.9	107.90	
Pelitinib (EKB-569)	100.63	85.52	76.13	>20	-0.31		83.39	
BMS-833923 (XL139)	-39.59	59.08	72.20	1.11	-0.37		68.77	
DBeQ	-36.11	88.88	73.45	1.19	-0.3		100.89	

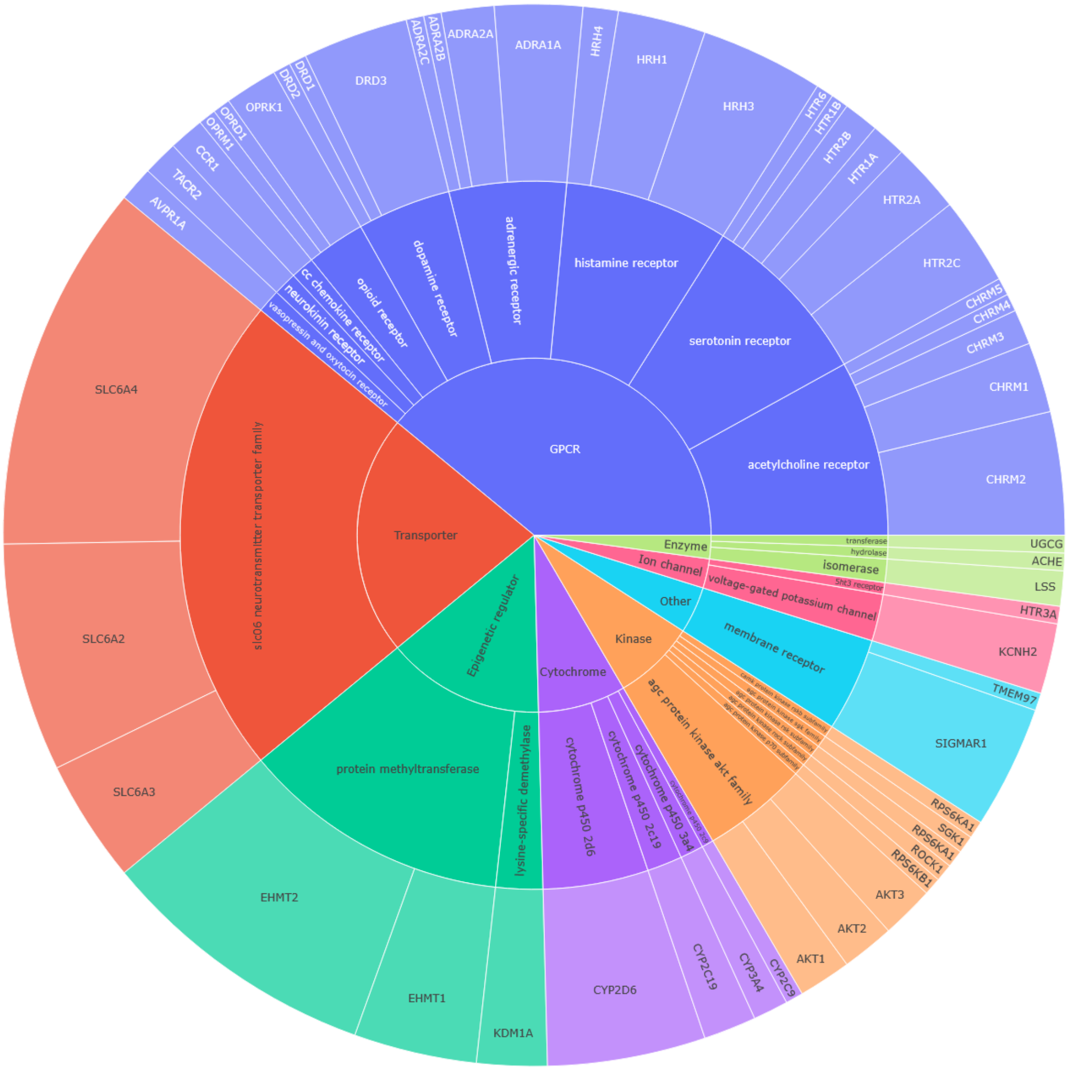

Figure S3. Distribution of cellular target for hit compounds identified in the primary screen for PLD induction in Vero-E6 and A549-ACE2. Annotation according to ChEBML v34. The 58 active compounds were searched into the ChEMBL database for identification of previously reported binding or functional assay experiments with other known targets. To achieve this, we used the pythonic functions provided by the KGG workflow (https://github.com/Fraunhofer-ITMP/kgg). The resulting output contains informtion about each compound's measured value (eg. Log Ki, IC50, AC50, etc.) for a specific target in a particular assay experiment (Supplementary file "Annotated_targets_to_PLD_hits").

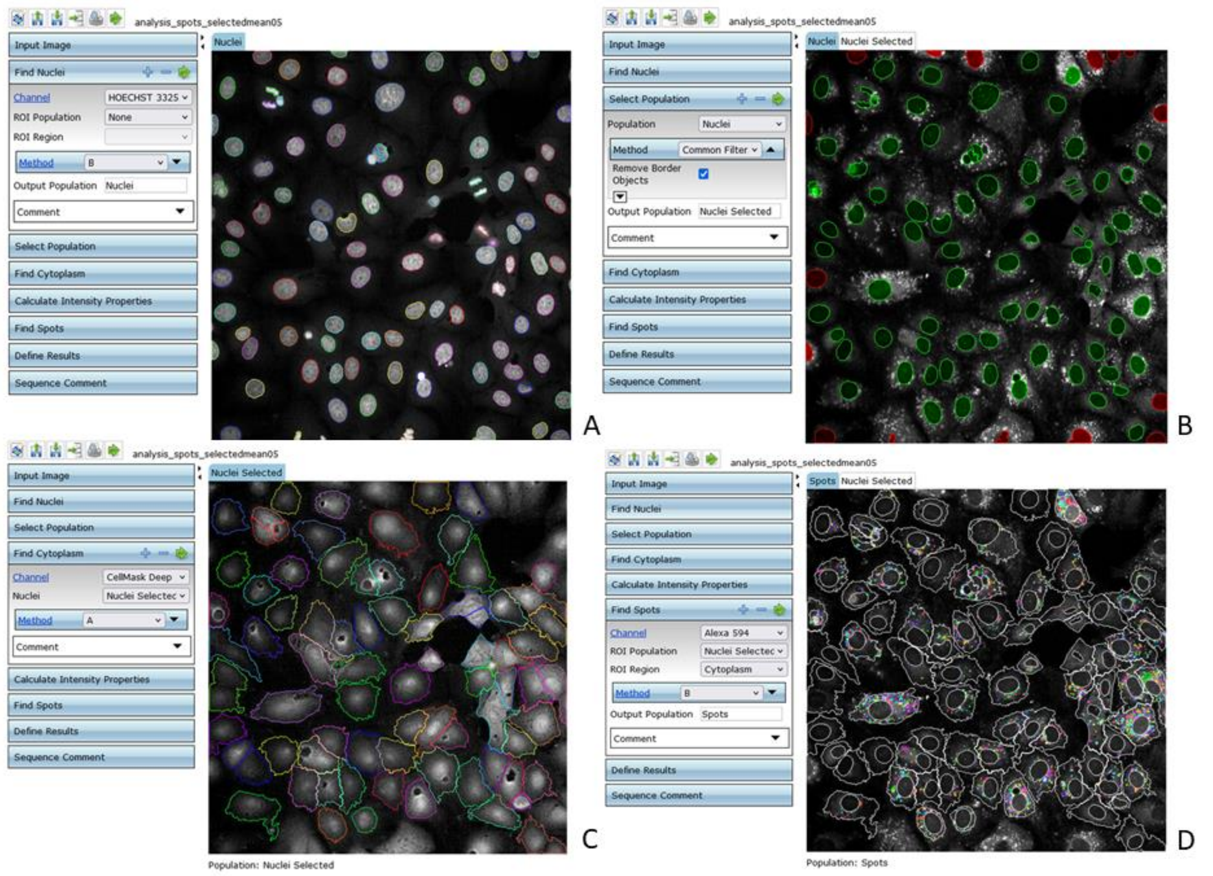

Figure S4: Workflow of DIPL data analysis in Columbus Image Analysis Software. Nuclei identification (A) Removement of border objects (nuclei are completely displayed) = "selected population" (B) Cytoplasm identification in the selected population (C) Calculation of DIPL signal in the cytoplasm: Identification of spots (D).

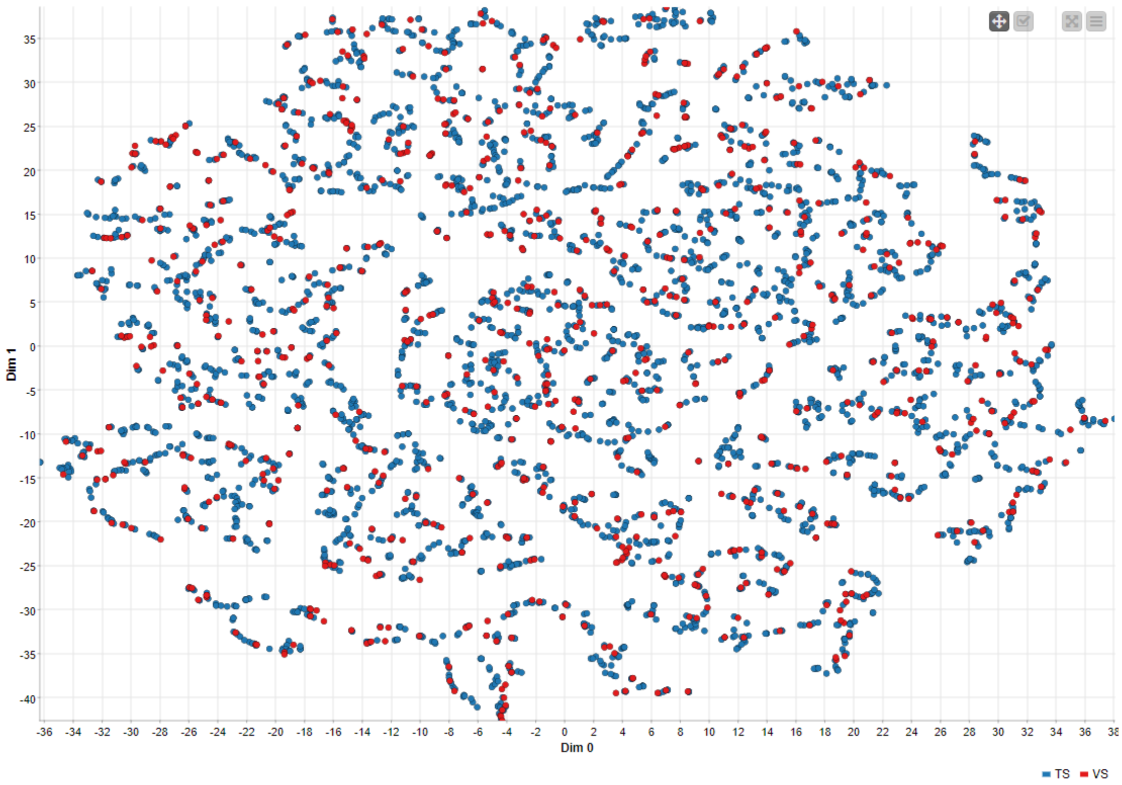

Figure S5: t-SNE plot visualizing training set and validation set. The dots in blue depict training set, and those in red depict validation. The homogeneous distribution of the chemicals (i.e no label specific clustering or pattern) indicate that the validation set is a representative of the training set data
